# Circular RNA hsa_circ_0008305 (circPTK2) inhibits TGF-β-induced epithelial-mesenchymal transition and metastasis by controlling TIF1γ in non-small cell lung cancer

**DOI:** 10.1186/s12943-018-0889-7

**Published:** 2018-09-27

**Authors:** Longqiang Wang, Xin Tong, Zhengyu Zhou, Shengjie Wang, Zhe Lei, Tianze Zhang, Zeyi Liu, Yuanyuan Zeng, Chang Li, Jun Zhao, Zhiyue Su, Cuijuan Zhang, Xia Liu, Guangquan Xu, Hong-Tao Zhang

**Affiliations:** 1 Soochow University Laboratory of Cancer Molecular Genetics, Medical College of Soochow University, 199 Ren’ai Road, Suzhou, 215123 Jiangsu China; 2 Department of Thoracic Surgery, The Second Affiliated Hospital of Harbin Medical University, 246 Xuefu Road, Harbin, 150086 Heilongjiang China; 3 Department of Respiratory Medicine, The First Affiliated Hospital of Soochow University, Medical College of Soochow University, Suzhou, 215006 Jiangsu China; 4 Department of Thoracic Surgery, The First Affiliated Hospital of Soochow University, Medical College of Soochow University, Suzhou, 215006 Jiangsu China; 5 Laboratory Animal Center, Medical College of Soochow University, Suzhou, 215123 Jiangsu China; 6 Department of Basic Medicine, Kangda College of Nanjing Medical University, Lianyungang, 222000 China; 7 Suzhou Key Laboratory for Molecular Cancer Genetics, Suzhou, 215123 Jiangsu China

**Keywords:** NSCLC, CircPTK2, TIF1γ, EMT, miR-429/miR-200b-3p

## Abstract

**Background:**

TGF-β promotes tumor invasion and metastasis through inducing epithelial-mesenchymal transition (EMT) in non-small cell lung cancer (NSCLC). Circular RNAs (circRNAs) are recognized as functional non-coding RNAs involved in human cancers. However, whether and how circRNAs contribute to TGF-β-induced EMT and metastasis in NSCLC remain vague. Here, we investigated the regulation and function of Circular RNA hsa_circ_0008305 (circPTK2) in TGF-β-induced EMT and tumor metastasis, as well as a link between circPTK2 and transcriptional intermediary factor 1 γ (TIF1γ) in NSCLC.

**Methods:**

Circular RNAs were determined by human circRNA Array analysis, real-time quantitative reverse transcriptase PCR and northern blot. Luciferase reporter, RNA-binding protein immunoprecipitation (RIP), RNA pull-down and fluorescence in situ hybridization (FISH) assays were employed to test the interaction between circPTK2 and miR-429/miR-200b-3p. Ectopic overexpression and siRNA-mediated knockdown of circPTK2, TGF-β-induced EMT, Transwell migration and invasion in vitro, and in vivo experiment of metastasis were used to evaluate the function of circPTK2. Transcription and prognosis analyses were done in public databases.

**Results:**

CircPTK2 and TIF1γ were significantly down-regulated in NSCLC cells undergoing EMT induced by TGF-β. CircPTK2 overexpression augmented TIF1γ expression, inhibited TGF-β-induced EMT and NSCLC cell invasion, whereas circPTK2 knockdown had the opposite effects. CircPTK2 functions as a sponge of miR-429/miR-200b-3p, and miR-429/miR-200b-3p promote TGF-β-induced EMT and NSCLC cell invasion by targeting TIF1γ. CircPTK2 overexpression inhibited the invasion-promoting phenotype of endogenous miR-429/miR-200b-3p in NSCLC cells in response to TGF-β. CircPTK2 overexpression significantly decreased the expression of Snail, an important downstream transcriptional activator of TGF-β/Smad signaling. In an in vivo experiment of metastasis, circPTK2 overexpression suppressed NSCLC cell metastasis. Moreover, circPTK2 expression was dramatically down-regulated and positively correlated with TIF1γ expression in human NSCLC tissues. Especially, circPTK2 was significantly lower in metastatic NSCLC tissues than non-metastatic counterparts.

**Conclusion:**

Our findings show that circPTK2 (hsa_circ_0008305) inhibits TGF-β-induced EMT and metastasis by controlling TIF1γ in NSCLC, revealing a novel mechanism by which circRNA regulates TGF-β-induced EMT and tumor metastasis, and suggesting that circPTK2 overexpression could provide a therapeutic strategy for advanced NSCLC.

**Electronic supplementary material:**

The online version of this article (10.1186/s12943-018-0889-7) contains supplementary material, which is available to authorized users.

## Background

Lung cancer is the leading cause of cancer-related deaths worldwide, and ~ 85% of all lung cancers are non-small cell lung cancer (NSCLC) [1, 2]. Despite improvement in therapeutic strategies, NSCLC patients yet exhibits poor prognosis [3]. This is predominantly attributed to tumor metastasis [4], suggesting that elucidation of the mechanisms underlying NSCLC metastasis is becoming a big challenge.

Transforming growth factor β (TGF-β) is highly expressed in NSCLCs [5–7]. Our previous study showed that TGF-β can promote epithelial-mesenchymal transition (EMT) and NSCLC cell invasion [8, 9]. In fact, there are compelling data that TGF-β/Smad signaling potently contributes to EMT and tumor metastasis in various human cancers [10, 11]. Recently, we have provided evidence that repression of transcriptional intermediary factor 1 γ (TIF1γ), a regulator of TGF-β/Smad signaling [12, 13], enhanced TGF-β-induced EMT in NSCLC cells [14]. In support of this, TIF1γ exerts its repressive activity on TGF-β/Smad signaling and plays an antagonistic role in TGF-β-induced EMT in mammary epithelial cells [11, 15, 16]. These data strongly suggest that TIF1γ functions as a tumor metastasis suppressor in human cancers, including NSCLC, by inhibiting TGF-β-induced EMT.

CircRNAs, a class of non-coding RNAs, are involved in gene regulation at both transcriptional and post-transcriptional levels [17]. Most of circRNAs are derived from a single exon or multiple exons and are detected in the cytoplasm [18, 19]. CircRNAs have been discovered to work as miRNA sponges [17, 20, 21]. The best-known ones so far include the recently identified circRNA, ciRS-7, which can efficiently tether miR-7, leading to reduced miR-7 activity and increased levels of oncogenic factors in cancer-associated signaling pathways [22]. Most recently, Hsiao et al. found that circCCDC66 may protect *MYC* mRNA from the attack of miRNA-33b and miR-93 to promote colon cancer growth and metastasis [23]; Han et al. reported that circMTO1 inhibits hepatocellular carcinoma progression by disrupting oncogenic miR-9 and promoting p21 expression [24]. However, the regulatory mechanisms of circRNAs in cancer need to be extensively validated [21]. Of more importance, hundreds of circRNAs were regulated in human mammary cells undergoing EMT [25], suggesting that certain circRNAs play important roles in TGF-β-induced EMT and thus influence cancer metastasis. RNA-binding protein Quaking (QKI) was identified to control biogenesis of > 30% of abundant circRNAs during EMT in response to TGF-β [25]. Furthermore, QKI is frequently down-regulated in NSCLC tissues and significantly associated with poorer prognosis [26]. These findings suggested that down-regulated circRNAs may be implicated in NSCLC progression, invasion and metastasis.

Taken together, we hypothesized that there may be several dysregulated circRNAs affecting TIF1γ activity and thereby promoting TGF-β-induced EMT and invasion in NSCLC. To test this, we first performed Human circRNA Array analysis in NSCLC cells before and after they underwent EMT in response to TGF-β, and identified 187 differentially expressed circRNAs. From the viewpoint of prediction, we focus on a down-regulated circRNA (hsa_circ_0008305 in circBase: http://www.circbase.org) produced from the *PTK2* gene, termed as circPTK2. Intriguingly, we further investigated the regulation and function of circPTK2 in TGF-β-induced EMT and tumor metastasis, as well as a link between circPTK2 and TIF1γ in NSCLC. Our findings show that circPTK2 suppresses TGF-β-induced EMT and tumor cell invasion by controlling TIF1γ in NSCLC, revealing a novel mechanism by which circRNA regulates TGF-β-induced EMT and tumor metastasis.

## Results

### TIF1γ and circPTK2 are down-regulated during TGF-β-induced EMT in NSCLC cells

**Fig. 1:**
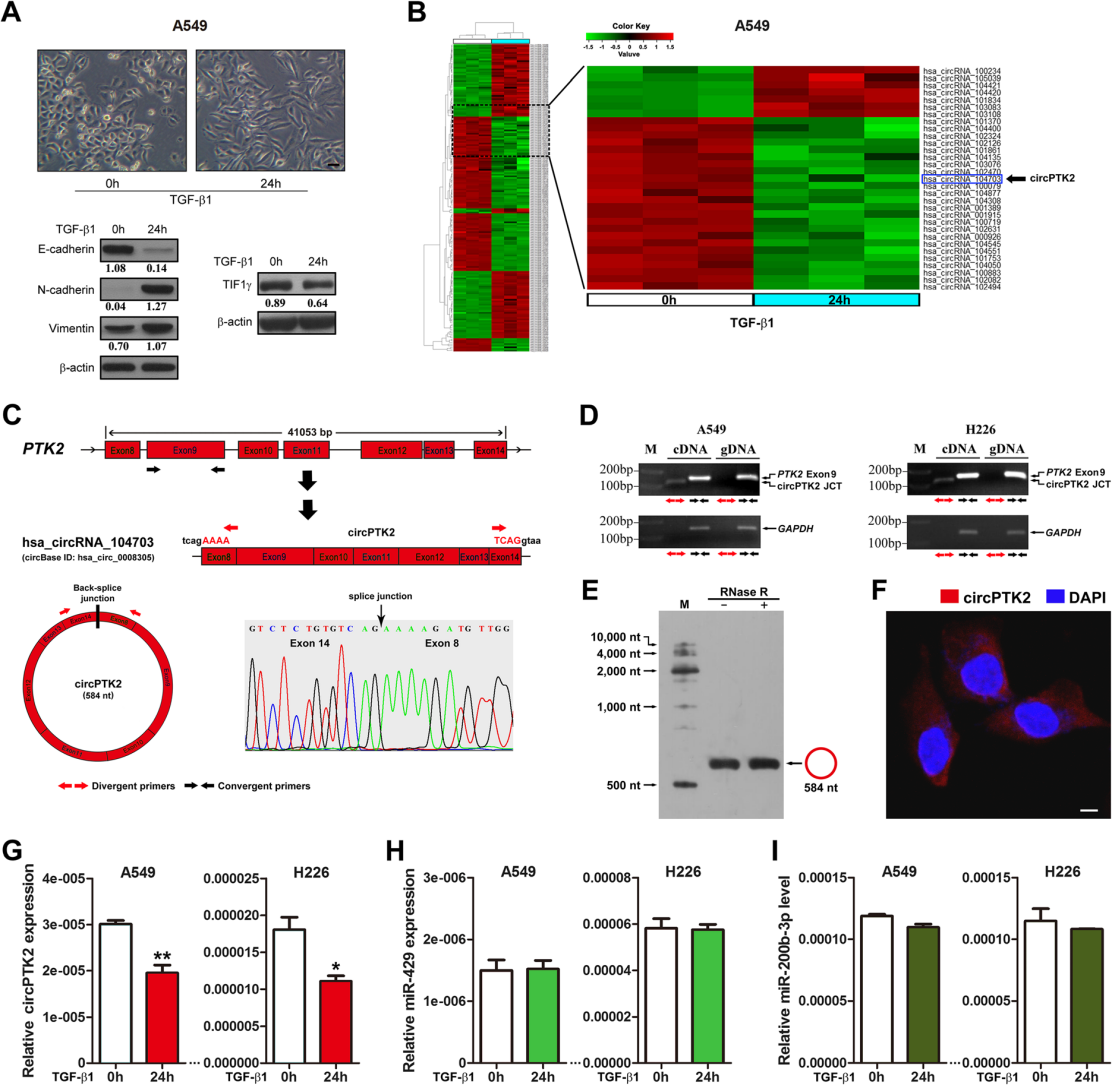
TIF1γ and circPTK2 are down-regulated during TGF-β-induced EMT in NSCLC cells. **a** A549 cells underwent epithelial-mesenchymal transition (EMT) after TGF-β1 (5 ng/ml) treatment for 24 h. Cell morphology was observed and photographed with a phase-contrast microscope (*upper*). Scale bar, 50 μm. The expression of EMT-related makers including E-cadherin, N-cadherin and Vimentin (*bottom left*), and TIF1γ protein (*bottom right*) were examined by western blot. β-actin was used as internal control. Densitometry values for each protein were normalized to β-actin and shown below the corresponding bands. **b** RNA from epithelial and mesenchymal A549 cells were subjected to Arraystar Human circRNA Array analysis as described in Methods. Hierarchical cluster analysis (heat map) of microarray data was used to show the significant expression of circRNAs when comparing mesenchymal cells with epithelial cells (*left*). Red and green denoted high and low expression, respectively. Each column represents a test sample and each row represents a circRNA. Each group (treated with TGF-β1 for 0 h or 24 h) was analyzed in triplicate. In a zoomed-in view of partial (*right*), the expression of circPTK2 (hsa_circRNA_104703) was indicated as an arrow. **c** The sketch of genomic locus of circPTK2 in *PTK2* gene. The expression of circPTK2 (circBase ID: hsa_circ_0008305) was validated by RT-PCR followed by sanger sequencing. Red arrows represent divergent primers, which are used to amplify the genome region of circPTK2 containing the back-splice junction site (JCT). **d** In A549 or H226 cells, divergent primers amplify circPTK2 JCT in cDNA but not in genomic DNA (gDNA), convergent primers amplify both circPTK2 JCT and linear *PTK2* Exon 9. *GAPDH* was used as linear control. Red and black arrows represent divergent and convergent primers, respectively. Divergent primers spanning circPTK2 JCT yield a product of 110 bp, while the convergent primers amplifying *PTK2* exon 9 yield a product of 141 bp. **e** Endogenous circPTK2 expression in A549 cells was validated by northern blots. RNase R was used to digest linear RNA. **f** Representative image of RNA fluorescence in situ hybridization for endogenous circPTK2 in A549 cells. Cell nuclei were counterstained with 4,6-diamidino-2-phenylindole (DAPI). Scale bar, 5 μm. **g** qRT-PCR analysis of circPTK2 expression in A549 and H226 cells treated with TGF-β1 for 24 h. Relative circPTK2 expression was determined with normalization against β-actin. **h, i** qRT-PCR analysis of miR-429/miR-200b-3p expression levels in A549 and H226 cells treated with TGF-β1 for 24 h. U6 was used as internal control. *P < 0.05; **P < 0.01

Public data suggest that TIF1γ is a tumor suppressor in NSCLC progression (Additional file 1: Figure S1A-D). Moreover, increased expression of TGF-β has been proved in NSCLC [5–7, 27] and TIF1γ knockdown promotes TGF-β-induced EMT in NSCLC cells [14]. To investigate whether TGF-β can down-regulate TIF1γ expression in EMT, we first examined TIF1γ expression in A549 cells before and after they had undergone EMT in response to TGF-β1. The TGF-β1-treated A549 cells were steadily mesenchymal with typical morphology and maker expression (Fig. 1a). Meanwhile, TIF1γ expression was significantly reduced in TGF-β-induced EMT of A549 cells (Fig. 1a). Next, RNA from epithelial and mesenchymal A549 cells was subjected to Arraystar Human circRNA Array analysis. We found that 88 circRNAs were up-regulated and 99 circRNAs were down-regulated when comparing mesenchymal A549 cells with epithelial A549 cells (≥ 1.5-fold; *P* < 0.05) (Fig. 1b and Additional file 2: Table S1). Using computational algorithms based on TargetScan/miRBase and miRanda, we predicted that circPTK2 (Arraystar ID: hsa_circRNA_104703) and *TIF1γ* 3’-UTR harbor the binding sites for miR-429/miR-200b-3p (Additional file 3: Figure S2A-C). Therefore, we focused on circPTK2 which was down-regulated 1.6-fold in TGF-β-induced EMT (Fig. 1b and Additional file 2: Table S1), and validated the characterization of circPTK2 in A549 and H226 cells (Fig. 1c-f). To confirm the microarray result, we performed qRT-PCR analysis and found that circPTK2 expression was significantly reduced in A549 and H226 cells treated with TGF-β1 for 24 h (Fig. 1g). Moreover, miR-429/miR-200b-3p levels were unchanged in A549 and H226 cells treated with TGF-β1 (Fig. 1h and i), which is supported by the findings that miR-200 family members were not altered by TGF-β1 in A549 cells [28]. Taken together, we deduced that circPTK2 may be positively associated with TIF1γ by acting as sponges of miR-429/miR-200b-3p in TGF-β-induced EMT of NSCLC cells.

### CircPTK2 binds directly to miR-429/miR-200b-3p in NSCLC cells

**Fig. 2:**
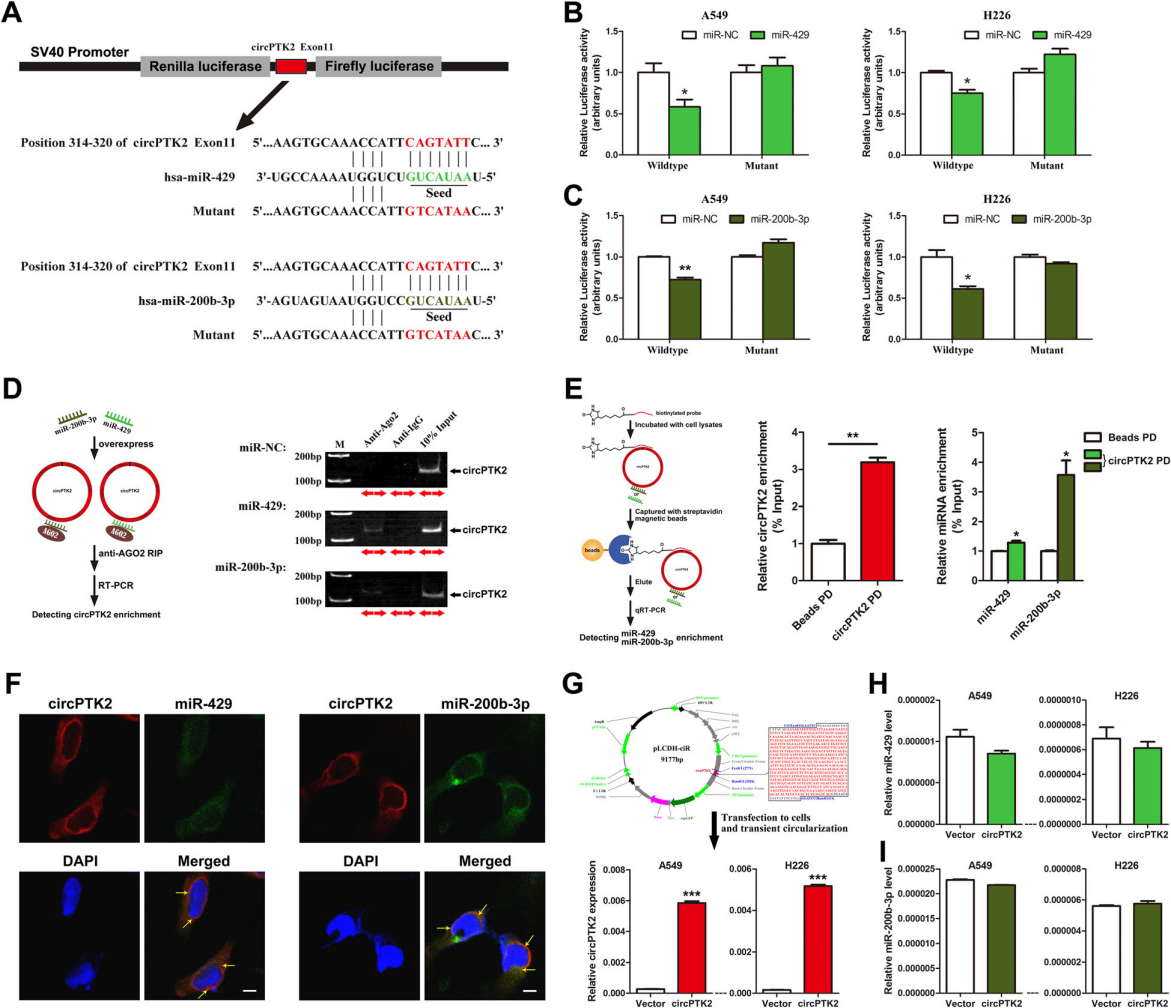
CircPTK2 binds directly to miR-429/miR-200b-3p in NSCLC cells. **a** Schematic description for the subcloning of the predicted miR-429/miR-200b-3p binding sites of circPTK2 exon11 in psiCHECK-2 luciferase vector. Predicted duplex formation between miR-429/miR-200b-3p and the wild-type/mutant of miR-429/miR-200b-3p binding sites of circPTK2 exon11 was shown. The entire subcloning sequences were listed in Additional file 4: Table S2. **b**, **c** Relative luciferase activity of the wild-type/mutant circPTK2 exon11 reporter gene in A549 and H226 cells transfected with miR-429/miR-200b-3p or negative control (miR-NC). Scrambled sequence was used as miR-NC. Relative *Renilla* luciferase activity was determined after normalizing against the firefly luciferase activity. **d** According to the flowchart outlining the experimental procedures (*left*), anti-AGO2 RIP was conducted in A549 cells transiently overexpressing miR-429/miR-200b-3p or miR-NC, and followed by RT-PCR and gel-staining analyses to detect circPTK2 enrichment (*right*). The 10% input was obtained as positive control before immunoprecipitation, and subjected to RT-PCR to confirm the presence of circPTK2. Anti-IgG antibody was used as a negative control. Red arrows represent divergent primers spanning the back-splice junction site of circPTK2. **e** According to the RNA pull-down flowchart (*left*), whole-cell lysates from A549 cells were incubated with biotinylated probes against circPTK2; after pull-down, endogenous circPTK2 (*middle*) and miR-429/miR-200b-3p (*right*) enrichments were detected by qRT-PCR. Results were presented as the percentage of pull-down to input. PD, pull-down. **f** Co-localization between circPTK2 (red) and miR-429/miR-200b-3p (green) was observed (arrowheads) by fluorescence in situ hybridization in A549 cells. Cell nuclei were counterstained with DAPI (blue). Scale bar, 5 μm. **g** CircPTK2 expression in A549 and H226 cells transiently overexpressing circPTK2. Cells were transiently transfected with pLCDH-circPTK2-copGFP(T2A)Puro lentiviral expression vector (*upper panel*) for 48 h or 72 h and then subjected to qRT-PCR analysis (*bottom panel*). The empty vector was served as negative control. In the upper panel, the subcloned sequence in quadrate box includes front circular frame, back circular frame of circRNA biogenesis (grey part) and full-length of 584-bp circPTK2 (red part). **h**, **i** Endogenous miR-429/miR-200b-3p levels in A549 and H226 cells transiently overexpressing circPTK2. *P < 0.05; **P < 0.01; ***P < 0.001

Endogenous circRNAs have been discovered to work as miRNA sponges [21, 29]. In view of the prediction that there is a shared binding site for miR-429/miR-200b-3p in each circPTK2 molecule (Additional file 3: Figure S2A), we performed the following experiments to verify whether circPTK2 binds to miR-429/miR-200b-3p in NSCLC cells. Firstly, we subcloned the circPTK2 region (Additional file 4: Table S2) containing miR-429/miR-200b-3p binding sites (wild type/mutant) into psiCHECK-2 luciferase vectors (Fig. 2a) and transiently cotransfected the reporter construct with miR-429/miR-200b-3p mimics into A549 and H226 cells. The luciferase activity assay showed that circPTK2 was bound by miR-429/miR-200b-3p (Fig. 2b and c). Secondly, given that miRNAs exert their biological functions in an AGO2-dependent manner [30], we performed anti-AGO2 RIP in A549 cells transiently overexpressing miR-429/miR-200b-3p. Endogenous circPTK2 pull-down by AGO2 was specifically enriched in A549 cells transfected with miR-429/miR-200b-3p (Fig. 2d), validating the direct binding of circPTK2 with miR-429/miR-200b-3p. Thirdly, RNA pull-down analysis demonstrated that endogenous miR-429/miR-200b-3p were significantly pulled down by biotinylated probes against circPTK2 (Fig. 2e), confirming the existence of circPTK2-miR-429/miR-200b-3p complexes. Fourthly, fluorescence in situ hybridization (FISH) experiments showed that circPTK2 and miR-429/miR-200b-3p were preferentially co-localized in the cytoplasm (Fig. 2f), supporting the direct interaction of circPTK2 with miR-429/miR-200b-3p. Furthermore, we transiently overexpressed more than 20-fold circPTK2 in A549 and H226 cells (Fig. 2g) and observed nonsignificant difference in miR-429/miR-200b-3p levels between cells overexpressing circPTK2 and control cells (Fig. 2h and i). Taken together, these results suggested that circPTK2 can function as a sponge for miR-429/miR-200b-3p in NSCLC cells.

### miR-429/miR-200b-3p represses TIF1γ expression by targeting 3’-UTR of *TIF1γ*

**Fig. 3:**
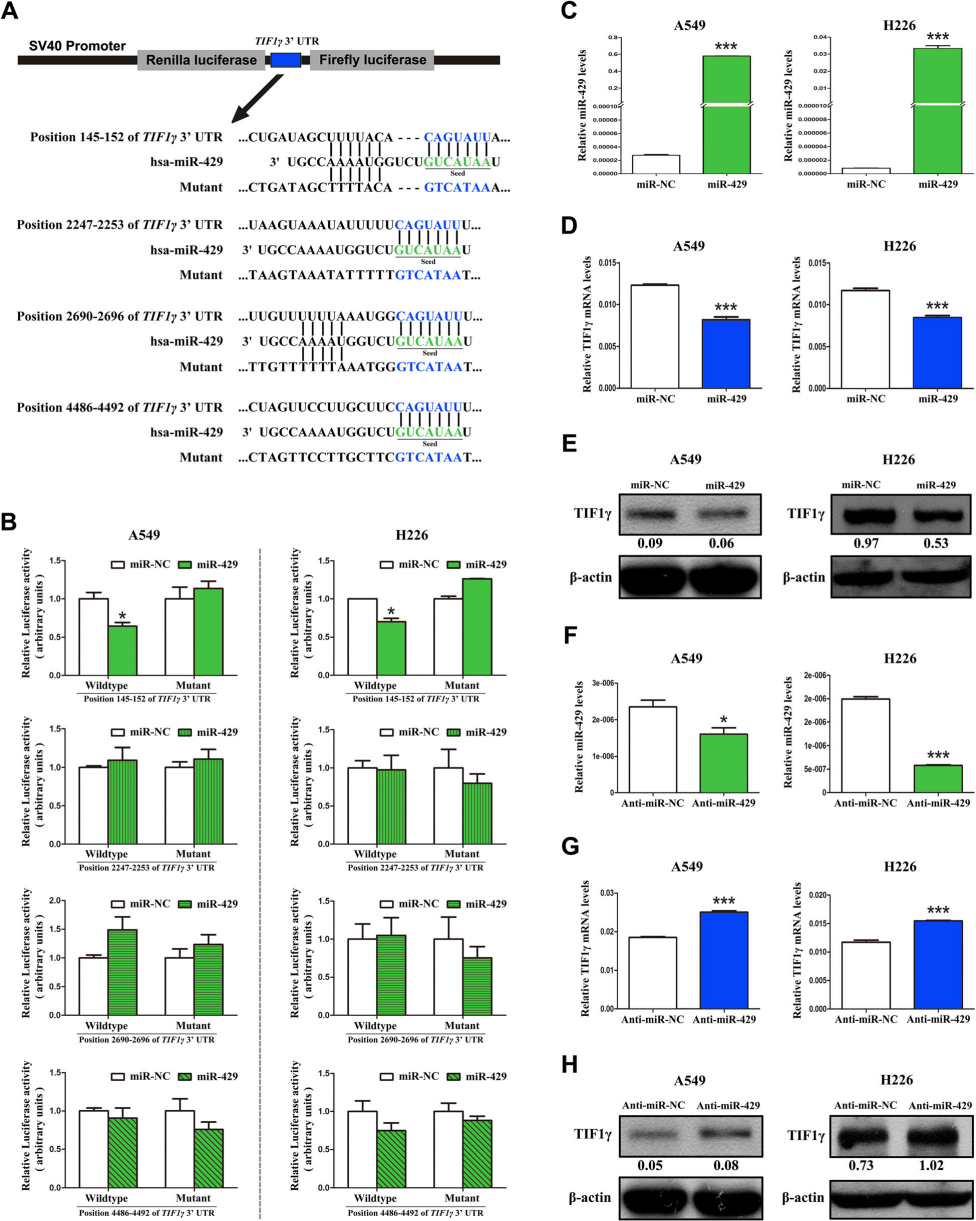
miR-429 represses TIF1γ expression by targeting 3’-UTR of *TIF1γ* transcript. **a** Schematic description for the subcloning of the predicted miR-429 binding sites of *TIF1γ* 3’-UTR in psiCHECK-2 luciferase vector. Predicted duplex formation between miR-429 and the wild-type/mutant of miR-429 binding sites was indicated. The entire subcloning sequences were listed in Additional file 4: Table S2. **b** Relative luciferase activity of the wild-type/mutant *TIF1γ* 3’-UTR reporter gene in A549 and H226 cells transfected with miR-429 or negative control (miR-NC). Scrambled sequence was used as miR-NC. Relative *Renilla* luciferase activity was determined after normalizing against the firefly luciferase activity. **c** qRT-PCR analysis of miR-429 expression levels in A549 and H226 cells transfected with miR-429 mimics or miR-NC. U6 was employed as internal control. **d**, **e** TIF1γ mRNA and protein expression in A549 and H226 cells transfected with miR-429 mimics or miR-NC. β-actin was used as internal control. **f** miR-429 expression levels in A549 and H226 cells transfected with miR-429 inhibitors (anti-miR-429) or negative control (anti-miR-NC). Scrambled sequence was used as anti-miR-NC. **g**, **h** TIF1γ mRNA and protein expression in A549 and H226 cells transfected with anti-miR-429 or anti-miR-NC. *P < 0.05; ***P < 0.001

Next, we predicted in silico that four different regions of *TIF1γ* 3’-UTR were putative targets of miR-429/miR-200b-3p (Additional file 3: Figure S2B and C). To test this, we subcloned the *TIF1γ* 3’-UTR regions (Additional file 4: Table S2) containing miR-429/miR-200b-3p binding sites (wild type/mutant) into psiCHECK-2 luciferase vectors (Fig. 3a and Additional file 5: Figure S3A) and transiently cotransfected the reporter construct with miR-429/miR-200b-3p mimics into A549 and H226 cells. The results showed that *TIF1γ* 3’-UTR was a target of miR-429/miR-200b-3p and the positions 145–152 and 2690–2696 of *TIF1γ* 3’-UTR were bona fide target sites of miR-429 and miR-200b-3p, respectively (Fig. 3b and Additional file 5: Figure S3B). Moreover, transiently overexpressed miR-429/miR-200b-3p (Fig. 3c and Additional file 5: Figure S3C) remarkably inhibited TIF1γ expression in A549 and H226 cells (Fig. 3d and e; Additional file 5: Figure S3D and E). In contrast, transient overexpression of miR-429/miR-200b-3p inhibitors (Fig. 3f and Additional file 5: Figure S3F) augmented TIF1γ expression in A549 and H226 cells (Fig. 3g and h; Additional file 5: Figure S3G and H). Taken together, these results demonstrated that miR-429/miR-200b-3p can inhibit TIF1γ expression by directly targeting the 3’-UTR of *TIF1γ* in NSCLC cells.

### CircPTK2 functions as sponge to protect TIF1γ from the attack of miR-429/miR-200b-3p

To investigate whether circPTK2 abolished endogenous miR-429/miR-200b-3p-mediated repression of TIF1γ, we administrated miR-429/miR-200b-3p mimics into A549 cells transiently overexpressing circPTK2 and examined TIF1γ expression. As illustrated in Additional file 6: Figure S4A and B, circPTK2 overexpression promoted TIF1γ expression while exogenous miR-429/miR-200b-3p mimics abrogated circPTK2 overexpression effect. Combined with the aforementioned results obtained in Figs. 2, 3, and Additional file 5: Figure S3, these findings confirmed that circPTK2 protected TIF1γ by sponging out miR-429/miR-200b-3p in NSCLC cells.

### miR-429/miR-200b-3p enhances TGF-β-induced EMT and invasion in NSCLC cells

**Fig. 4:**
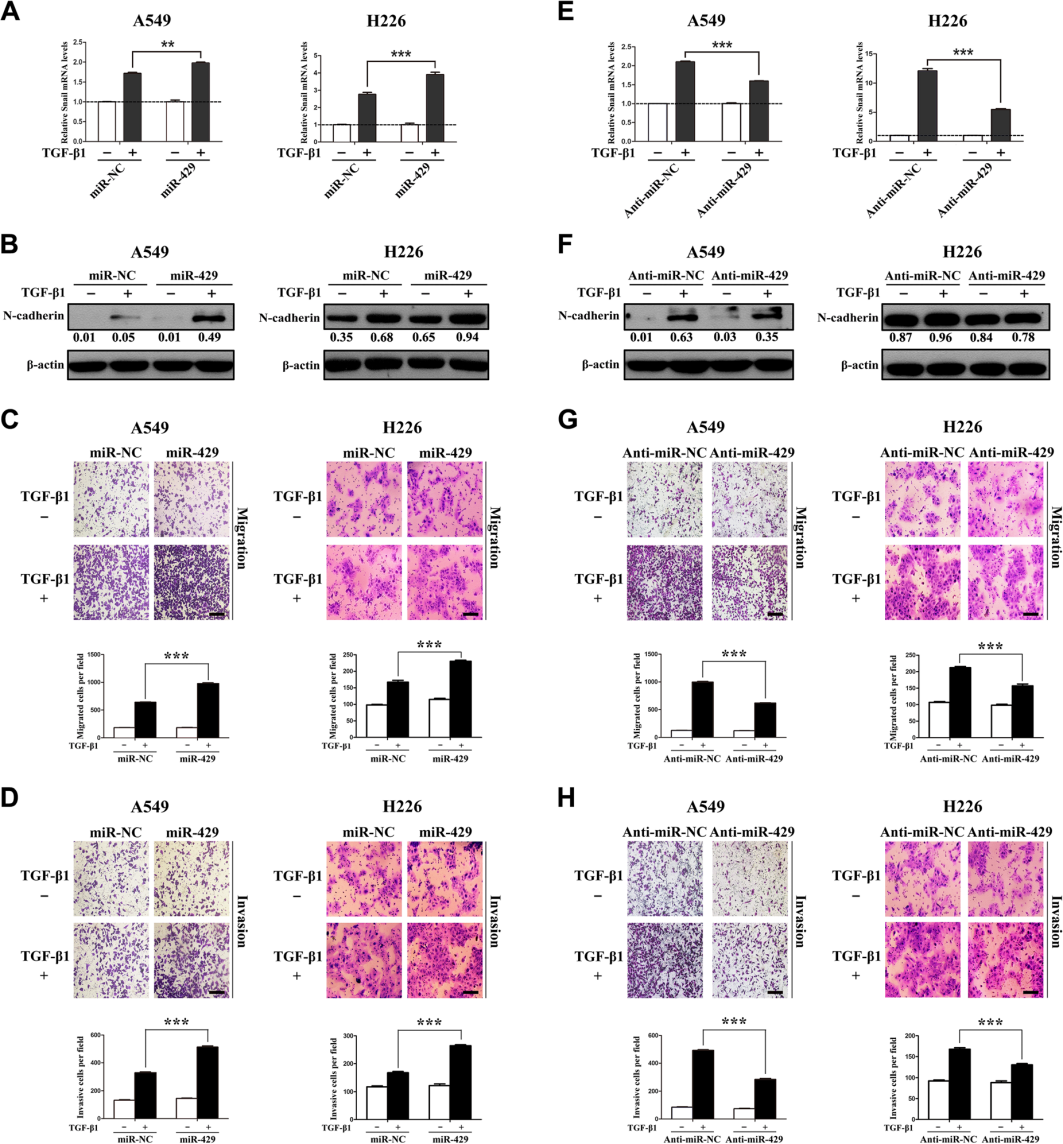
miR-429 enhances TGF-β-induced EMT and invasion in NSCLC cells. **a** After being serum-starved for 24 h, A549 and H226 cells transiently overexpressing miR-429 were treated with or without TGF-β1 (5 ng/ml) for 1 h and 2 h, respectively. *Snail* mRNA expression was quantified by qRT-PCR analysis. *Snail* mRNA level of the unstimulated cells was assigned the value 1, and the relative *Snail* mRNA expression in TGF-β1-stimulated cells was recalculated accordingly. **b** After being serum-starved for 24 h, A549 and H226 cells transiently overexpressing miR-429 were treated with or without TGF-β1 (5 ng/ml) for 24 h and 48 h, respectively. Western blot analysis was performed to examine the expression of N-cadherin, which was normalized to β-actin. **c** A549 and H226 cells transiently overexpressing miR-429 were treated as above and allowed to migrate through an 8-μM pore in transwells. Migrated cells were stained and counted in at least three light microscopic fields. Scale bar, 100 μm. **d** Cells were treated as above and allowed to invade through Matrigel-coated membrane in transwells. Invasive cells were stained and counted under a light microscope. Scale bar, 100 μm. **e** After being serum-starved for 24 h, A549 and H226 cells transiently overexpressing anti-miR-429 were treated with or without TGF-β1 (5 ng/ml) for 1 h and 2 h, respectively. qRT-PCR analysis was done to determine the relative *Snail* mRNA expression. **f** After being serum-starved for 24 h, A549 and H226 cells transiently overexpressing anti-miR-429 were treated with or without TGF-β1 (5 ng/ml) for 24 h and 48 h, respectively. N-cadherin expression was analyzed by western blot. **g** A549 and H226 cells transiently overexpressing anti-miR-429 were treated as above and allowed to migrate through an 8-μM pore in transwells. Migrated cells were stained and counted in at least three light microscopic fields. Scale bar, 100 μm. **h** Cells were treated as above and allowed to invade through Matrigel-coated membrane in transwells. Invasive cells were stained and counted under a light microscope. Scale bar, 100 μm. **P < 0.01; ***P < 0.001

Our previous findings demonstrated that TIF1γ knockdown promotes TGF-β-induced EMT and NSCLC cell invasion [14]. Considering the present findings that miR-429/miR-200b-3p repressed TIF1γ expression, we thus hypothesized that miR-429/miR-200b-3p may lead to the same phenotype caused by TIF1γ knockdown in A549 and H226 cells. Upon TGF-β1 stimulation, A549 and H226 cells overexpressing miR-429/miR-200b-3p showed higher expression of *Snail* mRNA and N-cadherin compared with the cells transfected with miR-NC (Fig. 4a and b; Additional file 7: Figure S5A and B). Furthermore, miR-429/miR-200b-3p increased TGF-β-induced migratory and invasive abilities of A549 and H226 cells (Fig. 4c and d; Additional file 7: Figure S5C and D). In contrast, in the presence of TGF-β1, A549 and H226 cells transfected with miR-429/miR-200b-3p inhibitor showed lower expression of *Snail* mRNA and N-cadherin (Fig. 4e and f; Additional file 7: Figure S5E and F), and displayed a reduction in migration and invasion (Fig. 4g and h; Additional file 7: Figure S5G and H). Taken together, the results indicated that miR-429/miR-200b-3p can enhance TGF-β-induced EMT and NSCLC cell invasion.

### CircPTK2 overexpression enhances TIF1γ expression and inhibits TGF-β-induced EMT and NSCLC cell invasion

**Fig. 5:**
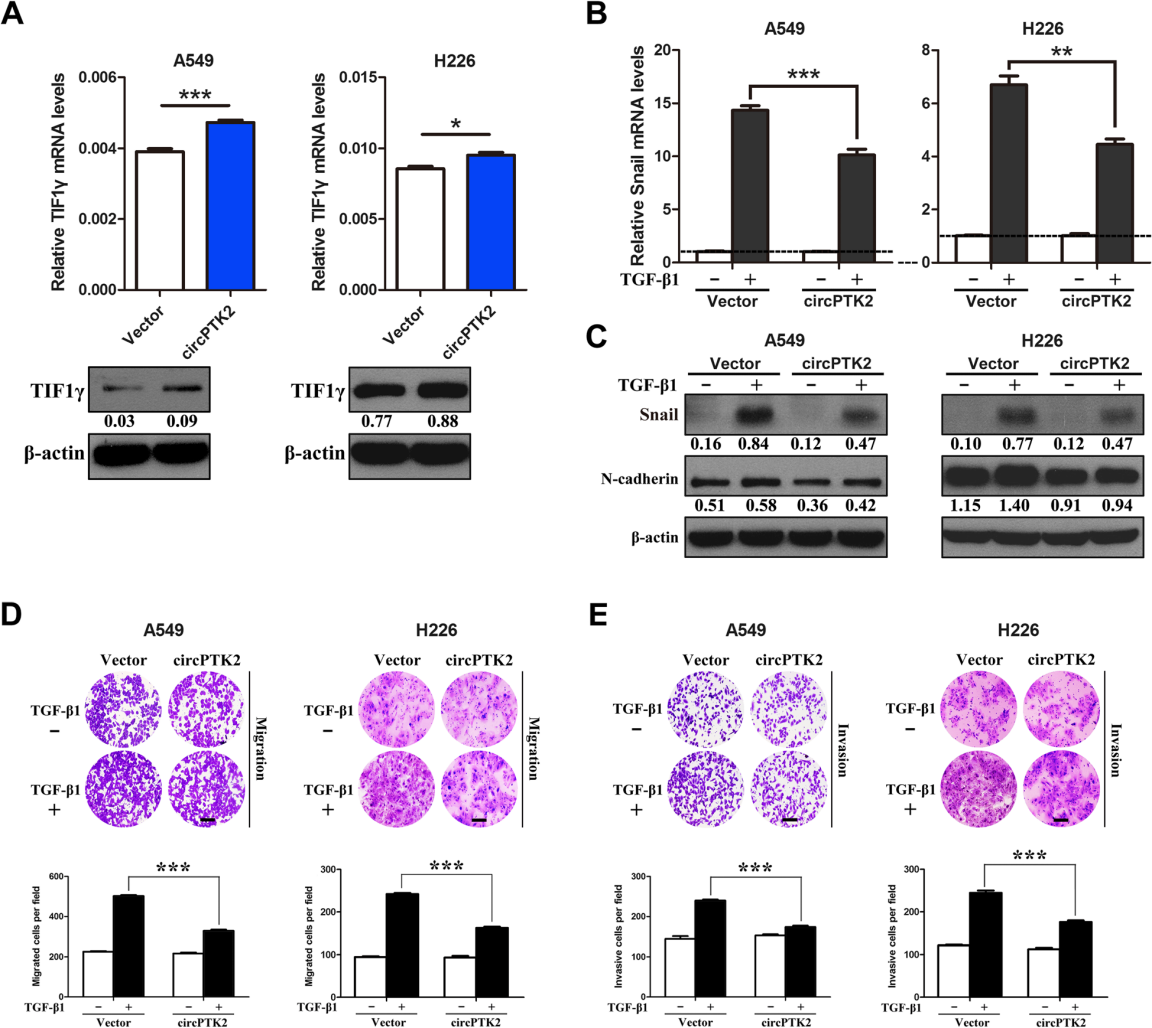
Overexpression of circPTK2 enhances TIF1γ expression and inhibits TGF-β-induced EMT and invasion of NSCLC cells in vitro. **a** TIF1γ mRNA and protein levels in A549 and H226 cells transiently overexpressing circPTK2. Relative TIF1γ expression was determined with normalization against β-actin. **b** After being serum-starved for 24 h, A549 and H226 cells transiently overexpressing circPTK2 were treated with or without TGF-β1 (5 ng/ml) for 1 h and 0.5 h, respectively. *Snail* mRNA expression was quantified by qRT-PCR analysis. *Snail* mRNA level of the unstimulated cells was assigned the value 1, and the relative *Snail* mRNA expression in TGF-β1-stimulated cells was recalculated accordingly. **c** A549 and H226 cells transiently overexpressing circPTK2 were serum-starved for 24 h and then treated with or without TGF-β1 (5 ng/ml) for 24 h and 48 h, respectively. Snail and N-cadherin protein levels were determined by western blot. β-actin was used as internal control. **d**, **e** A549 and H226 cells transiently overexpressing circPTK2 were treated as above and subjected to the transwell migration and invasion assays. Migrated and invasive cells were stained and counted in at least three light microscopic fields. Scale bar, 100 μm. *P < 0.05; **P < 0.01; ***P < 0.001

We next examined whether circPTK2 influenced TIF1γ expression and TGF-β-induced EMT and invasive phenotypes in NSCLC cells. As a result, transient overexpression of circPTK2 (Fig. 2g) significantly augmented TIF1γ expression in A549 and H226 cells (Fig. 5a). Moreover, upon TGF-β1 stimulation, A549 and H226 cells transiently overexpressing circPTK showed lower expression of Snail and N-cadherin compared with control cells (Fig. 5b and c). Comparably, circPTK2 significantly suppressed TGF-β-induced migratory and invasive abilities of A549 and H226 cells (Fig. 5d and e). The phenotype of circPTK2 overexpression (Fig. 5d and e) copied the invasion-suppressing phenotype of miR-429/miR-200b-3p inhibitors in A549 and H226 cells (Fig. 4g and h; Additional file 7: Figure S5G and H) and circPTK2 overexpression inhibited the invasion-promoting phenotype of endogenous miR-429/miR-200b-3p in A549 cells in response to TGF-β (Additional file 6: Figure S4C and D). Collectively, the results indicated that circPTK2 overexpression can promote TIF1γ expression and inhibit TGF-β-induced EMT and NSCLC cell invasion by abrogating the effects of miR-429/miR-200b-3p.

### CircPTK2 knockdown inhibits TIF1γ expression and promotes TGF-β-induced EMT and NSCLC cell invasion

To verify the aforementioned roles of circPTK2 in vitro, we designed siRNA specifically targeting circPTK2 back-splice junction (JCT) to knockdown circPTK2 function (Additional file 8: Figure S6A, left). Transfection with this siRNA effectively silenced circPTK2 levels in A549 and H226 cells (Additional file 8: Figure S6A, right) but had no effect on linear *PTK2* mRNA levels (Additional file 8: Figure S6B). CircPTK2 knockdown remarkably attenuated TIF1γ expression in A549 and H226 cells (Additional file 8: Figure S6C). On TGF-β1 stimulation, circPTK2-silenced A549 and H226 cells showed higher expression of Snail and N-cadherin than those of control cells (Additional file 8: Figure S6D). Moreover, circPTK2 knockdown significantly increased TGF-β-induced migratory and invasive abilities of A549 and H226 cells (Additional file 8: Figure S6E and F). Taken together, these results revealed that circPTK2 knockdown can inhibit TIF1γ expression and promote TGF-β-induced EMT and NSCLC cell invasion.

### CircPTK2 overexpression attenuates NSCLC cell metastasis in vivo

**Fig. 6:**
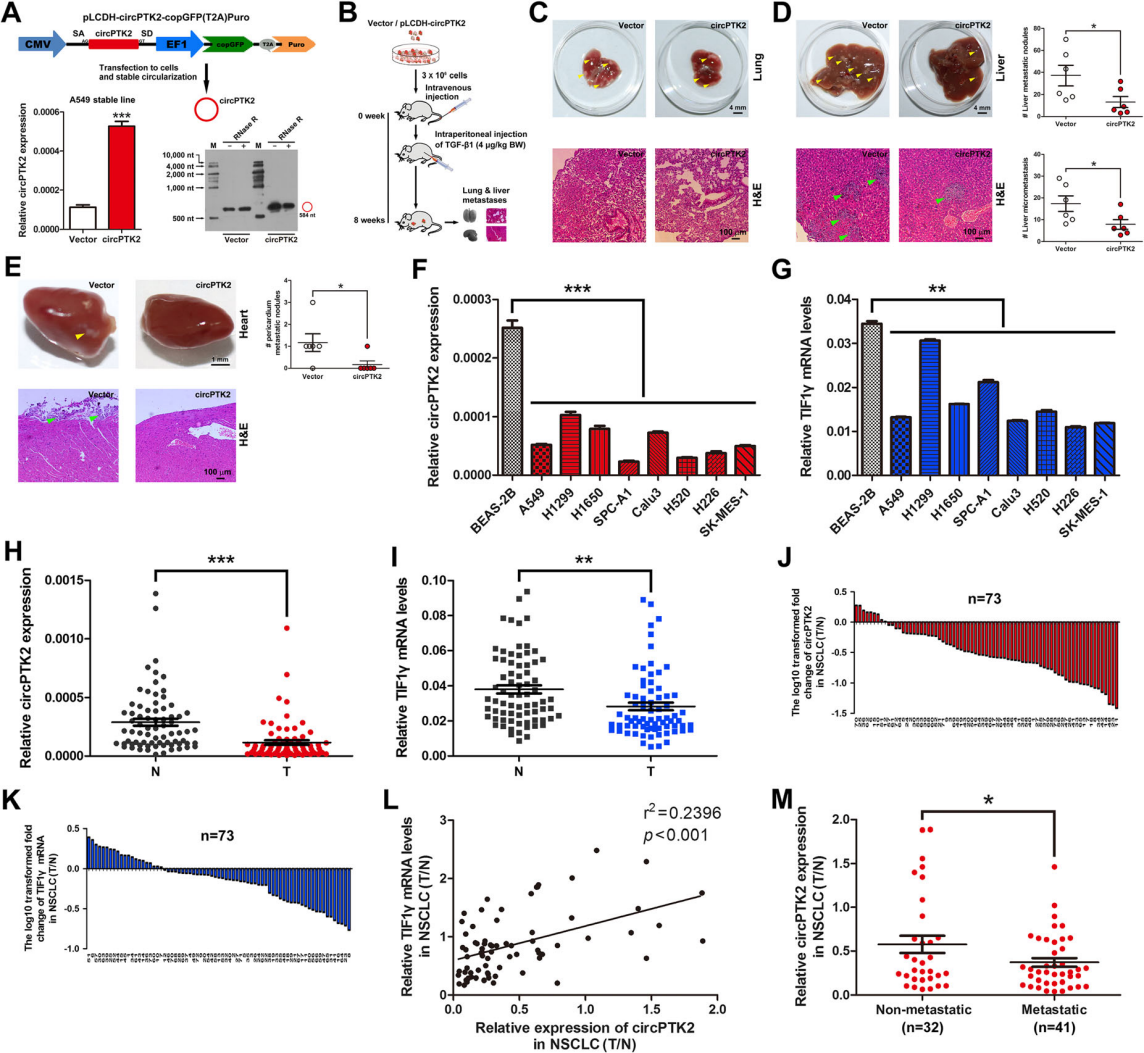
CircPTK2 overexpression attenuates NSCLC cell metastasis in vivo, and circPTK2 levels were lower in metastatic NSCLC tissues than non-metastatic counterparts. **a** CircPTK2 expression in A549 cells stably overexpressing circPTK2. A549 stable cell line overexpressing circPTK2 was generated as described in Methods. pLCDH-circPTK2-copGFP(T2A)Puro lentiviral expression vector (*upper*) was used to stably overexpress circPTK2. The empty vector was served as negative control. CircPTK2 expression was determined by qRT-PCR (*bottom left*). CircPTK2 expression in circPTK2-overexpressed A549 cells was determined using northern blots. RNase R was used to digest linear RNA (*bottom right*). **b** Schematic flowchart of the in vivo metastasis experiments with A549 cells stably transfected with pLCDH-circPTK2 or vector (i.v.) and TGF-β1 (i.p.) injected into BALB/c nude mice (*n* = 6 mice per group in circPTK2 + TGF-β1 and vector + TGF-β1). **c** Representative images showing metastatic nodules established in lung taken from the mice injected with circPTK2-overexpressed A549 cells or vector control cells (*upper*). Scale bar, 4 mm. Haematoxylin and eosin (H&E) staining was performed for histological confirmation of metastasizing tumor cells in lung (*bottom*). Scale bar, 100 μm. **d** Gross view of metastatic nodules developed in liver (*upper left*) and dot plots showing the number of metastatic nodules in liver (*upper right*, n = 6 mice per group). Scale bar, 4 mm. Microscopic images of H&E staining for liver metastases (*bottom left*) and the distribution of the number of metastases in per section of liver (*bottom right*, *n* = 6 mice per group). Scale bar, 100 μm. Yellow and green arrowheads indicate metastatic nodules and micrometastases. **e** Representative images indicating metastatic nodules developed in pericardium (*upper*, n = 6 mice per group) and H&E staining of heart (*bottom*). Scale bar, 1 mm or 100 μm. **f**, **g** CircPTK2 and *TIF1γ* mRNA expression levels in human lung epithelial and NSCLC cells. β-actin was used as internal control. Each qRT-PCR analysis was performed in triplicate. **h**, **i** qRT-PCR analysis of circPTK2 and *TIF1γ* mRNA levels in 73 human NSCLC tissues and paired noncancerous lung tissues. Mean values are indicted by solid bars, and values are expressed as mean ± SEM. T, NSCLC tissues; N, paired noncancerous lung tissues. **j**, **k** Relative expression of circPTK2 and *TIF1γ* mRNA in 73 paired NSCLC tissues. *Y*-axis represents the log_10_ transformed fold change of T/N expression ratios of circPTK2 and *TIF1γ* mRNA. The number of each specimen is shown below *x*-axis. **l** Correlation between circPTK2 level and *TIF1γ* mRNA expression in 73 paired NSCLC tissues. *X* and *y* axes represent the T/N expression ratios of circPTK2 and *TIF1γ* mRNA, respectively. **m** Relative expression (T/N) of circPTK2 in metastatic (*n* = 41) and non-metastatic (*n* = 32) NSCLC tissues. Metastatic tissues were from NSCLC patients with lymph node metastasis or distant metastasis and non-metastatic tissues were from NSCLC patients without any metastasis, respectively. *P < 0.05; **P < 0.01; ***P < 0.001

To further investigate the role of circPTK2 overexpression in NSCLC cell metastasis in vivo*,* we first established A549 cells where circPTK2 can stably overexpress. About 5-fold high expression of circPTK2 was consistently exhibited in A549 cells infected with the packaged lentivirus containing pLCDH-circPTK2-copGFP(T2A)Puro plasmids; Northern blot analysis confirmed the presence of circPTK2 overexpression in A549 cells (Fig. 6a). Then, circPTK2-overexpressed and control A549 cells were injected i.v. into BALB/c nude mice through the tail vein, and TGF-β1 was injected i.p. post cell inoculation (Fig. 6b). Eight weeks post-inoculation, we euthanized the mice and surgically resected lungs, livers and hearts for evaluation of metastases and histology. As expected, the mice injected with circPTK2-overexpressed A549 cells or vector control cells effectively exhibited lung metastasis (Fig. 6c). Importantly, the mice injected with circPTK2-overexpressed A549 cells developed less metastatic nodules in livers and pericardia compared with those injected with control cells (Fig. 6d and e). Moreover, we monitored lung metastasis of luciferase-tagged A549 cells in vivo by the bioluminescent imaging at day 42 post-inoculation, and confirmed the metastasis-suppressing function of circPTK2 (Additional file 9: Figure S7A-G). Collectively, the in vivo experiment of metastasis shows that circPTK2 overexpression inhibits NSCLC cell metastasis in vivo.

### CircPTK2 expression was lower in metastatic NSCLC tissues than non-metastatic counterparts

We determined that circPTK2 and *TIF1γ* mRNA levels were significantly lower in 8 NSCLC cell lines than human lung normal epithelial cells (Fig. 6f and g). To further evaluate whether these data are reflected in patients, we detected circPTK2 and *TIF1γ* mRNA levels in 73 NSCLC and paired noncancerous lung tissues. NSCLC tissues showed a significant reduction in their expression (87.7% for circPTK2 and 69.9% for *TIF1γ* mRNA, respectively) when compared with noncancerous lung tissues (Fig. 6h-k). Among 64 NSCLC tissues with low circPTK2 expression, 49 tumors (76.6%) showed low TIF1γ expression (Fig. 6j and k; Additional file 10: Table S3); In contrast, of 7 NSCLC tissues with high circPTK2 expression, 4 tumors (57.1%) presented high TIF1γ expression (Fig. 6j and k; Additional file 10: Table S3). Moreover, the ratio of circPTK2 expression (T/N) was positively correlated with that of *TIF1γ* mRNA level (T/N) in NSCLC tissues (*P* < 0.001; Fig. 6l). Importantly, circPTK2 was significantly lower in metastatic NSCLC tissues as compared to their non-metastatic counterparts (Fig. 6m). Therefore, a close link between circPTK2 and TIF1γ was established in NSCLC, supporting the notion that circPTK2 functions as a tumor suppressor through regulating TIF1γ.

## Discussion

To date, whether and how circRNAs contribute to TGF-β-induced EMT in NSCLC remains elusive. In the present study, we reveal that circPTK2 inhibits TGF-β-induced EMT by up-regulating TIF1γ in NSCLC, and establish a novel mechanistic role of circPTK2-TIF1γ axis in regulating TGF-β-induced EMT (Additional file 11: Figure S8).

TIF1γ (alias, TRIM33/RFG7/PTC7/Ectodermin), a regulator of TGF-β/Smad signaling, acts as an “antagonist” by ubiquitinating Smad4 or a “complementary agonist” by competing with Smad4 to regulate TGF-β/Smad signaling [12, 13]. TIF1γ has been proved to contribute to multiple malignancies [16, 31–33], and TIF1γ is essential for regulating TGF-β signaling [34, 35] and EMT [11]. Our previous studies show that TIF1γ expression is frequently reduced in NSCLC and TIF1γ repression enhances TGF-β-induced EMT and NSCLC cell invasion [14, 36]. Knockdown of TIF1γ increases the expression of Snail [14], an important downstream transcriptional activator of TGF-β/Smad signaling [9]. Here, we show that reduced TIF1γ is not just associated with poor survival of NSCLC patients but also positively correlated with circPTK2 expression in NSCLC tissues. Moreover, circPTK2 expression were significantly reduced in NSCLC cells and circPTK2 overexpression augmented TIF1γ expression in NSCLC cells. Thus, for the first time, we established a link between TIF1γ and circPTK2 in NSCLC. Actually, circPTK2 is produced from the *PTK2* gene, which spans seven of its exons. We validated the characterization of circPTK2 in NSCLC cells (Fig. 1c-f). It has been well documented that exon-intron or intron-derived circRNAs promote their parent gene transcription in cell nucleus, but exon-derived circRNAs do not affect the expression of their parent genes [29]. In the present study, circPTK2 derived from multiple exons of *PTK2* was detected in the cytoplasm (Fig. 1f) and circPTK2 did not influence its parent gene *PTK2* expression (Additional file 8: Figure S6B). To the best of our knowledge, circPTK2 is a circular RNA which function has not yet been defined in human cancers. In this study, we identified that circPTK2 can function as a sponge for miR-429/miR-200b-3p to counter degradation of TIF1γ. Moreover, miR-429/miR-200b-3p promoted TGF-β-induced EMT and cell invasion by inhibiting TIF1γ in NSCLC cells. These data suggests that circPTK2 has potential functional role in TGF-β-induced EMT and NSCLC metastasis.

Although circRNAs’ expression is often low, circRNAs are emerging as oncogenic stimuli or tumor suppressors in cancer [37]. To date, the mechanistic roles of circRNAs in TGF-β-induced EMT are poorly understood in NSCLC. Therefore, we primarily performed circRNA microarray analysis and identified that circPTK2 was significantly reduced during TGF-β-induced EMT of NSCLC cells. Moreover, circPTK2 overexpression inhibited TGF-β-induced EMT and NSCLC cell invasion, whereas its knockdown had the opposite effect. Especially, circPTK2 was significantly lower in metastatic NSCLC tissues than non-metastatic counterparts, supporting the roles of circPTK2 in NSCLC cell invasion in vitro. Furthermore, the in vivo experiment of metastasis showed that circPTK2 overexpression suppressed NSCLC cell metastasis. Collectively, these results suggested that circPTK2 may function as a tumor metastasis suppressor by controlling TGF-β signaling activity.

There are multiple and diverse molecular mechanisms of TGF-β-induced EMT in human cancers [38, 39]. In fact, it is not surprising because various molecules participate in TGF-β signaling, which is a potent inducer of EMT. Unexceptionally, long non-coding RNAs (lncRNAs) and microRNAs (miRNAs) are also implicated in TGF-β signaling and EMT [40–42]. As exemplified, miR-145 and miR-203 repressed SMAD3, a downstream effector in canonical TGF-β/Smad signaling, then inhibited TGF-β-induced EMT in NSCLC cells [43]. In the present study, miR-429/miR-200b-3p acted as invasion-promoting miRNAs to promote TGF-β-induced EMT in NSCLC cells. Our results are different from the previous findings where miR-429/miR-200b-3p was recognized as anti-metastatic miRNAs in glioma and renal cell carcinoma [44, 45]. However, Lang et al. reported that miR-429 promoted the metastasis of NSCLC cells [46], supporting our results obtained in NSCLC cells. This can be explained by the idea that miRNAs may exert distinct roles depending on the cellular context, which is probably attributed to the availability of specific targets or downstream effectors [47]. Moreover, the invasion-promoting phenotype of miR-429/miR-200b-3p overexpression was copied by knockdown of TIF1γ, which promoted TGF-β-induced EMT in NSCLC cells [14]. In fact, our data and a public data set (GSE36681) suggests that miR-429/miR-200b exerts a tumor-promoting role in NSCLCs (Additional file 12: Table S4 and Additional file 13: Figure S9A-F). It has been proposed that TGF-β induces EMT by driving the expression of ZEB transcription factors, which in turn inhibit miR-200 family members via a double-negative feedback loop [48]. However, on TGF-β1 stimulation, miR-429/miR-200b-3p levels were still unchanged in time-independent manner, albeit a dynamic alteration of ZEB1/ZEB2 expression in A549 cells (Additional file 14: Figure S10A-D). In support of this, Zhang et al. reported that miR-200 family members were not altered by TGF-β1 in A549 cells [28]. More importantly, we here provided the first evidence that TGF-β promotes EMT via circRNAs in NSCLC, albeit circPTK2 may be one of several circRNAs affecting TGF-β-induced EMT. Interestingly, TGF-β was able to down-regulate circPTK2 expression but failed to alter miR-429/miR-200b-3p levels in NSCLC cells. This result is consistent with that circPTK2 is frequently reduced in NSCLC tissues (Fig. 6h and j), where increased expression of TGF-β has been detected [6, 7, 27]. More recently, Conn et al. identified that formation of > 30% of abundant circRNAs was regulated by QKI during TGF-β-mediated EMT in human mammary cells [25]. Zong et al. reported that QKI is frequently reduced in NSCLC [26]. The two investigations on QKI might provide an explanation for why TGF-β diminished circPTK2 expression in NSCLC cells. Interestingly, QKI plays an important role in TGF-β-mediated downregulation of circPTK2 (Additional file 15: Figure S11A-D).

More interestingly, another circRNA hsa_circ_0003221 (also named after circPTK2), with a spliced sequence length of 625 nt in circBase (http://www.circbase.org), has been reported to promote the proliferation and migration of bladder cancer cells [49]. Although hsa_circ_0003221 and hsa_circ_0008305 are derived from the same parent gene *PTK*, they exert different functions. In fact, this is not surprising because they could have distinct mechanisms depending on the cellular context or downstream target molecules.

In summary, our findings show that circPTK2 (hsa_circ_0008305) inhibits TGF-β-induced EMT through regulating TIF1γ and NSCLC cell metastasis, as well as establish a positive relationship between TIF1γ and circPTK2 in NSCLC, revealing a novel mechanism by which circRNA regulates TGF-β-mediated EMT and tumor metastasis, and suggesting that overexpression of circPTK2 could provide a therapeutic strategy for advanced NSCLC.

## Methods

### Cell lines and cell culture

Human lung normal epithelial cell BEAS-2B, and NSCLC cells A549, H1299, H1650, SPC-A1, and Calu3 (lung adenocarcinoma cells) and H226, H520, and SK-MES-1 (lung squamous carcinoma cells) from Cell Bank of Chinese Academy of Sciences were cultured in RPMI 1640 medium (HyClone, South Logan, UT, USA) supplemented with penicillin/streptomycin, L-glutamine and 10% fetal bovine serum (FBS, Invitrogen, Carlsbad, CA, USA) at 37 °C in a humidified atmosphere with 5% CO_2_. A549 and H226 cells were induced by TGF-β1 to undergo EMT as per our description [9, 14].

### Tissue samples

Seventy-three fresh NSCLC tissues and paired adjacent noncancerous lung tissues (Additional file 10: Table S3) were collected after informed consent from patients in the First Affiliated Hospital of Soochow University. Histological and pathological diagnostics for NSCLC patients were evaluated based on the Revised International System for Staging Lung Cancer. Patients received neither chemotherapy nor radiotherapy before tissue sampling. As listed in Additional file 10: Table S3, metastatic tissues (*n* = 41) were from NSCLC patients with local lymph node metastasis (T_1-4_ N_1-2_ M_0_) or distant organ metastasis (T_1-4_N_any_M_1_), and non-metastatic tissues (*n* = 32) were from NSCLC patients without any metastasis (T_1-4_N_0_M_0_). The samples were snap-frozen in liquid nitrogen and stored at − 80 °C before RNA extraction. This study was approved by the Ethics Committee of Soochow University.

### Real-time quantitative reverse transcriptase PCR (qRT-PCR)

RNA was isolated using TRIzol (Thermo Fisher Scientific, Carlsbad, CA, USA). cDNA synthesis and qRT-PCR analysis were performed as described by us [50] with some modification. Primers are listed in Additional file 16: Table S5. U6 levels were used to normalize miR-429/miR-200b-3p expression. β-actin was endogenous control for *TIF1γ* mRNA, *Snail* mRNA, *PTK2* mRNA and circPTK2. Relative expression of each RNA was determined using the ΔΔ*C*_*t*_ method. Each qRT-PCR analysis was done in triplicate.

### Western blot analysis

Cells were lysed and subjected to western blot analysis as described by us [51]. Antibodies were as follows: mouse anti-TIF1γ (Santa Cruz Biotechnology, Santa Cruz, CA, USA), mouse anti-E-cadherin, anti-N-cadherin and anti-Vimentin (BD Biosciences, San Jose, CA, USA), mouse anti-Snail (Cell Signaling Technology, Danvers, MA, USA), mouse anti-β-actin and anti-mouse secondary antibodies (Santa Cruz Biotechnology). Molecular sizes of TIF1γ, E-cadherin, N-cadherin, Vimentin, Snail and β-actin proteins shown on the immunoblots are 150kD, 120kD, 130kD, 57kD, 29kD and 43kD, respectively. Each experiment was carried out in triplicate.

### CircRNA microarray analysis

Total RNA from A549 cells treated without and with TGF-β1 was used for Arraystar Human circRNA Array (Arraystar Inc., Rockville, MD, USA). CircRNA microarray analysis was performed as described [52]. CircRNAs (fold change ≥1.5 and *P*-value < 0.05) were considered to be differentially expressed between two groups. Each group (cells treated with TGF-β1 for 0 h or 24 h, respectively) was analyzed in triplicate.

### Northern blot with denaturing agarose gels

Digoxin-labeled DNA probes (351 nt), spanning the back-splice junction of circPTK2, were prepared from cDNA using PCR DIG Probe Synthesis Kit (Roche, Mannheim, Germany). PCR primers were as follows: 5’-GAATATGGCTGACCTAATAGA-3′ (forward); 5’-ACACTTGAAGCATTCCTTATC-3′ (reverse).

Total RNA (15 μg) denatured in formaldehyde was resolved on 1% agarose-formaldehyde gel and transferred onto a Hybond-N^+^ nylon membrane (GE Healthcare, Buckinghamshire, UK). Membranes were crosslinked, pre-hybridized in DIG Easy Hyb (Roche), and hybridized with DIG-labeled DNA probes overnight. After stringent washing, the membranes was incubated with alkaline phosphatase (AP)-conjugated anti-DIG antibodies (Roche). Immunoreactive bands were visualized using chemiluminescent substrate CSPD (Roche) followed by exposure to X-ray film.

### Prediction of miRNA targets

CircRNA/miRNA interaction was predicted with miRNA target prediction software (Arraystar’s home-made) based on TargetScan and miRanda. TargetScan (Release 7.1, http://www.targetscan.org) or miRBase (Release 21, http://www.mirbase.org) were employed to identify the miRNA targeting sites in *TIF1γ* 3’-UTR.

### Luciferase reporter assay

A series of constructs containing *TIF1γ* 3’-UTR and circPTK2 exon11 were generated using psiCHECK2 dual luciferase vector (Promega, Madison, WI, USA). Different fragments (Additional file 4: Table S2) were directly synthesized (GENEWIZ Inc., Suzhou, China), subcloned into the psiCHECK-2 vector to create various constructs. Each construct was subsequently cotransfected with miR-429 mimic (5’-UAAUACUGUCUGGUAAAACCGU-3′) or miR-200b-3p mimic (5’-UAAUACUGCCUGGUAAUGAUGA-3′) and a negative control (miR-NC, 5’-UUCUCCGAACGUGUCACGUTT-3′) into A549 and H226 cells. All the transient transfections, including miR-429 inhibitor (5’-ACGGUUUUACCAGACAGUAUUA-3′) or miR-200b-3p inhibitor (5’-UCAUCAUUACCAGGCAGUAUUA-3′) and anti-miR-NC (5’-CAGUACUUUUGUGUAGUACAA-3′), were performed using Lipofectamine 2000 (Invitrogen). After 48 h, cells were harvested, and luciferase activities were determined by the Dual-Luciferase Reporter Assay Kit (Promega). Results are presented as relative *Renilla* luciferase activities, which are normalized to firefly luciferase activities. Each experiment was performed in triplicate.

### RNA-binding protein immunoprecipitation (RIP) assay

RIP assay was performed using EZ-Magna RIP Kit (Millipore, Billerica, MA, USA). The AGO2-RIP experiments were performed in A549 cells transiently overexpressing miR-429/miR-200b-3p or miR-NC. Briefly, cells were lysed using RIP lysis buffer with proteinase and RNase inhibitors (Millipore), and the RIP lysates were incubated with RIP buffer containing magnetic beads conjugated with human anti-Ago2 antibody or nonspecific mouse IgG antibody (Millipore). Each immunoprecipitate was digested with proteinase K, and the immunoprecipitated RNAs were subjected to RT-PCR and gel-staining analyses to detect circPTK2 enrichment. Each RIP assay was repeated three times.

### RNA pull-down analysis

RNA pull-down analysis was performed as previously described [53] with some modification. Briefly, the RIP lysates from A549 cells were incubated with biotin (Bio)-labeled oligonucleotide probes against circPTK2 (**Bio**-5’-TTAAACCAACATCTTTTCTGACACAGAGACGGCG-3′, RiboBio, Guangzhou, China) for 2 h at 25 °C. CircPTK2/miRNA complexes were captured with Streptavidin-coupled Dynabeads (Invitrogen). CircPTK2/miRNA/beads complexes were incubated with RIP wash buffer (Millipore) containing proteinase K for 1 h at 25 °C. CircPTK2 and miR-429/miR-200b-3p in the pull-down were determined using qRT-PCR analysis. The retrieved circPTK2 or miR-429/miR-200b-3p were evaluated as the percentage of pull-down to input. Each experiment was performed in triplicate.

### Fish

Cells were cultured on coverslips, fixed and permeablized as previously described by us [14]. Subsequently, the coverslips were hybridized in hybridization buffer (Geneseed Biotech, Guangzhou, China) with digoxin (Dig) and biotin (Bio)-labeled single-stranded DNA probes at 37 °C overnight. Digoxin-labeled probes (**Dig**-5’-CATCTTTTCTGACACAGAGACGGCG-3′-**Dig**) specific to circPTK2 back-splice region and biotin-labeled probes against miR-429/miR-200b-3p (for miR-429, **Bio**-5’-ACGGTTTTACCAGACAGTATTA-3’-**Bio**; for miR-200b-3p, **Bio**-5’-TCATCATTACCAGGCAGTATTA-3’-**Bio**) were prepared (Geneseed Biotech). The signals were detected by Cy3-conjugated anti-digoxin and FITC-conjugated anti-biotin antibodies (Jackson ImmunoResearch Inc., West Grove, PA, USA). Cell nuclei were counterstained with 4,6-diamidino-2-phenylindole (DAPI). Finally, the images were obtained on a Zeiss LSM 700 confocal microscope (Carl Zeiss, Oberkochen, Germany). Each experiment was performed three times.

### Establishment of NSCLC cells transiently and stably overexpressing circPTK2

To establish A549 and H226 cell lines transiently overexpressing circPTK2, we subcloned full-length of 584-bp circPTK2 into a pLCDH-ciR lentiviral expression vector (Geneseed Biotech) to generate pLCDH-circPTK2-copGFP(T2A)Puro construct. The subcloned sequence containing front circular frame (SA), back circular frame (SD) of circRNA biogenesis and full-length of circPTK2, 5’-TGAAATATGCTATCTTACAG-circPTK2-GTGAATATATTTTTTCTTGA-3′, was directly synthesized (GENEWIZ Inc.). Cells was transiently transfected with the construct using Lipofectamine 2000. The empty vector was used as negative control. After transfection for 48 or 72 h, cells were harvested for additional more experiments.

To generate A549 cells stably overexpressing circPTK2, we cotransfected the above-mentioned construct or empty vector with packaging plasmids psPAX2 and pMD2.G (Geneseed Biotech) into HEK 293 T cells using Lipofectamine 2000 (Invitrogen). After HEK 293 T cells were cultured for 48 h, the packaged lentiviruses were harvested. A549 cells were infected with the virus and cultured for 3 days. Finally, A549 cells were selected with 0.5 μg/ml of puromycin (Sigma-Aldrich, St. Louis, MO, USA) for in vivo experiments of metastasis.

### RNA interference for circPTK2 knockdown

CircPTK2 was specifically knockdown using siRNA (si-circPTK2, 5’-GUGUCAGAAAAGAUGUUGGUU-3′), which was designed by CircInteractome (http://circinteractome.nia.nih.gov) and synthesized (GenePharma, Shanghai, China) to target circPTK2 back-splice junction. Scramble siRNA (5’-CACAGUCAAAAGAUGUUGGUU-3′) was used as a negative control. A549 and H226 cells were transfected with 100 pmol of siRNA using Lipofectamine 2000 (Invitrogen). After 48 or 72 h, cells were harvested for qRT-PCR analysis of circPTK2 and linear *PTK2* mRNA expression or for other experiments.

### Transwell migration and invasion assays

Transwell assays were conducted to evaluate cell migration and invasion abilities as described by us [14]. Briefly, A549 and H226 cells transiently overexpressing miR-429/miR-200b-3p or circPTK2 were incubated with TGF-β1 (5 ng/ml) in Transwell plates (BD Biosciences) for 24 h and 48 h. Then cells were allowed to migrate through an 8-μM pored membrane or invade through Matrigel-coated membrane. Migrated and invasive cells were stained and counted under a light microscope. Transwell assays were done in triplicate.

### In vivo experiments of metastasis

Female BALB/c nude mice (4–6 weeks, 18–20 g) were purchased from the Laboratory Animal Center of Soochow University, and were bred and maintained in specific pathogen-free conditions. Mice were divided into two groups, including circPTK2 overexpression group and control group (6 mice per group). CircPTK2-overexpressed and control A549 cells (3 × 10^6^ cells/mouse) in PBS were intravenously (i.v.) injected into the tail vein of mice. TGF-β1 (4 μg/kg bodyweight) was injected intraperitoneally (i.p.) every 5th day post cell inoculation as previously described [27] to facilitate TGF-β-induced cancer cell invasion. Fifty-six days post-inoculation, the mice were sacrificed and their lung, liver and heart tissues were histologically analyzed with H&E staining for the presence of metastasizing tumor cells. Before H&E staining, the number of metastatic nodules established in lung, liver and pericardium was counted. To monitor tumor cells metastasized to lung, bioluminescent imaging was performed using an IVIS® Spectrum in vivo imaging system (Caliper Life Sciences, Hopkinton, MA, USA). Approximately 15 min before imaging, the mice were injected i.p. with D-luciferin sodium salt (Yeasen Biotech, Shanghai, China) in PBS (15 mg/ml) at a dose of 150 mg/kg bodyweight. Following air anaesthesia with isoflurane, live images were acquired using photography and photons emitted from active luciferase within a region of interest (ROI) were quantified using Living Image® 4.0 software (measured in photons/sec/cm^2^/steradian). Animal studies were approved by the Ethics Committee of Soochow University.

### Statistical analysis

Difference between two groups was assessed using paired or unpaired *t* test (two-tailed). Pearson’s correlation test was used to determine the association between two groups. Results were presented as mean ± SEM. *P* values of < 0.05 were considered significant. Statistical analyses were performed using GraphPad Prism 5.02 software (GraphPad, San Diego, CA, USA).

## Conclusions

In conclusion, our findings show that circPTK2 (hsa_circ_0008305) inhibits TGF-β-induced EMT and metastasis by controlling TIF1γ in NSCLC, revealing that circPTK2 has an important role in regulating TGF-β-induced EMT and tumor metastasis, and suggesting a rationale for therapeutically upregulating circular RNAs in patients with advanced NSCLC.

## Additional files

Additional file 1: **Figure S1.** Reduced TIF1γ is expressed in NSCLC tissues and associated with poor survival of NSCLC patients. (A, B) Data regarding *TIF1γ* mRNA expression in lung adenocarcinoma, squamous cell carcinoma tissues and normal lung tissues from several study groups in Oncomine database (http://www.oncomine.org). (C, D) Kaplan-Meier survival curves for 85 patients with lung adenocarcinoma (AdC) and 71 patients with lung squamous cell carcinoma (SqC). Primary data were taken from GSE30219 and TCGA in Kaplan-Meier Plotter (http://www.kmplot.com). (TIF 2402 kb)

Additional file 2: **Table S1.** Arraystar Human circRNA Array analysis of A549 cells treated with TGF-β1. Among 187 differentially expressed circRNAs (fold change ≥1.5, *P*-value < 0.05 and FDR < 0.05), 88 circRNAs were up-regulated and 99 circRNAs were down-regulated in A549 cells after TGF-β1 treatment for 24 h. CircRNA ID: encoded in circBase (http://www.circbase.org). P-value: estimated by paired t-test. FDR: false discovery rate, calculated from Benjamini Hochberg FDR. Fold change: the absolute ratio (no log scale) of normalized intensities between two conditions (treated with TGF-β1 vs. treated without TGF-β1). (DOC 195 kb)

Additional file 3: **Figure S2.** The in silico prediction of the interaction between miR-429/miR-200b-3p and circPTK2 or *TIF1γ* 3’-UTR. (A) The interaction of circPTK2 and miR-429/miR-200b-3p was predicted with miRNA target prediction software (Arraystar’s home-made) based on TargetScan and miRanda. (B, C) The target interaction between miR-429/miR-200b-3p and *TIF1γ* 3’-UTR was in silico predicted by TargetScan (Release 7.1 http://www.targetscan.org)/miRBase (Release 21, http://www.mirbase.org). Four different sites (positions 145–152, 2247–2253, 2690–2696 and 4486–4492) of *TIF1γ* 3’-UTR were predicted to be targets of miR-429/miR-200b-3p. (TIF 6394 kb)

Additional file 4: **Table S2.** Sequences for construction of luciferase reporter plasmids containing predicted miR-429 and miR-200b-3p target sites in *TIF1γ* 3’-UTR and circPTK2. (DOC 38 kb)

Additional file 5: **Figure S3.** miR-200b-3p inhibits TIF1γ expression by targeting 3’-UTR of *TIF1γ* transcript. (A) Schematic description for the subcloning of the predicted miR-200b-3p binding sites of *TIF1γ* 3’-UTR in psiCHECK-2 luciferase vector. Predicted duplex formation between miR-200b-3p and the wild-type/mutant of miR-200b-3p binding sites was indicated. The entire subcloning sequences were listed in Additional file 4: Table S2. (B) Relative luciferase activity of the wild-type/mutant *TIF1γ* 3’-UTR reporter gene in A549 and H226 cells transfected with miR-200b-3p or negative control (miR-NC). Scrambled sequence was used as miR-NC. Relative *Renilla* luciferase activity was determined after normalizing against the firefly luciferase activity. (C) qRT-PCR analysis of miR-200b-3p expression levels in A549 and H226 cells transfected with miR-200b-3p mimics or miR-NC. U6 was employed as internal control. (D, E) TIF1γ mRNA and protein expression in A549 and H226 cells transfected with miR-200b-3p mimics or miR-NC. β-actin was used as internal control. Densitometry values for TIF1γ protein were normalized to β-actin and shown below the corresponding bands. (F) miR-200b-3p expression levels in A549 and H226 cells transfected with miR-200b-3p inhibitors (anti-miR-200b-3p) or negative control (anti-miR-NC). Scrambled sequence was used as anti-miR-NC. (G, H) TIF1γ mRNA and protein expression in A549 and H226 cells transfected with anti-miR-200b-3p or anti-miR-NC. *P < 0.05; **P < 0.01; ***P < 0.001. (TIF 5059 kb)

Additional file 6: **Figure S4.** CircPTK2 abolishes endogenous miR-429/miR-200b-3p-mediated repression of TIF1γ and inhibits TGF-β-induced invasion of NSCLC cells. (A) *TIF1γ* mRNA expression in A549 cells transiently overexpressing circPTK2 or empty vector in the presence or absence of miR-429/miR-200b-3p mimics. OE, overexpression. (B) TIF1γ protein levels in A549 cells transiently overexpressing circPTK2 in the above-mentioned condition. Densitometry values for TIF1γ protein were normalized to β-actin and indicated below the corresponding bands. (C, D) A549 cells overexpressing circPTK2 and miR-429/miR-200b-3p mimics were serum-starved for 24 h, and then were subjected to Transwell migration and invasion assays in the presence or absence of TGF-β1 described as Methods. Migrated and invasive cells were stained and counted in at least three light microscopic fields. Scale bar, 100 μm. *P < 0.05; **P < 0.01; ***P < 0.001. (TIF 14297 kb)

Additional file 7: **Figure S5.** miR-200b-3p promotes TGF-β-induced EMT and invasion in NSCLC cells. (A) After being serum-starved for 24 h, A549 and H226 cells transiently overexpressing miR-200b-3p were treated with or without TGF-β1 (5 ng/ml) for 1 h and 2 h, respectively. *Snail* mRNA expression was quantified by qRT-PCR analysis. *Snail* mRNA level of the unstimulated cells was assigned the value 1, and the relative *Snail* mRNA expression in TGF-β1-stimulated cells was recalculated accordingly. (B) After being serum-starved for 24 h, A549 and H226 cells transiently overexpressing miR-200b-3p were treated with or without TGF-β1 (5 ng/ml) for 24 h and 48 h, respectively. Western blot analysis was performed to examine the expression of N-cadherin, which was normalized to β-actin. (C) A549 and H226 cells transiently overexpressing miR-200b-3p were treated as above and allowed to migrate through an 8-μM pore in transwells. Migrated cells were stained and counted in at least three light microscopic fields. Scale bar, 100 μm. (D) Cells were treated as above and allowed to invade through Matrigel-coated membrane in transwells. Invasive cells were stained and counted under a light microscope. Scale bar, 100 μm. (E) After being serum-starved for 24 h, A549 and H226 cells transiently overexpressing anti-miR-200b-3p were treated with or without TGF-β1 (5 ng/ml) for 1 h and 2 h, respectively. qRT-PCR analysis was done to determine the relative *Snail* mRNA expression. (F) After being serum-starved for 24 h, A549 and H226 cells transiently overexpressing anti-miR-200b-3p were treated with or without TGF-β1 (5 ng/ml) for 24 h and 48 h, respectively. N-cadherin expression was analyzed by western blot. (G) A549 and H226 cells transiently overexpressing anti-miR-200b-3p were treated as above and allowed to migrate through an 8-μM pore in transwells. Migrated cells were stained and counted in at least three light microscopic fields. Scale bar, 100 μm. (H) Cells were treated as above and allowed to invade through Matrigel-coated membrane in transwells. Invasive cells were stained and counted under a light microscope. Scale bar, 100 μm. *P < 0.05; ***P < 0.001. (TIF 5920 kb)

Additional file 8: **Figure S6.** CircPTK2 knockdown inhibits TIF1γ expression and promotes TGF-β-induced EMT and invasion of NSCLC cells in vitro. (A) *Left panel*, a siRNA targeting circPTK2 JCT (si-circPTK2) was designed to specifically knockdown circPTK2. *Right panel*, A549 and H226 cells were transfected with si-circPTK2 and siRNA negative control (si-NC). qRT-PCR was performed to detect circPTK2 expression in the siRNA-transfected cells. (B) Linear *PTK2* mRNA expression in circPTK2-silenced A549 and H226 cells. (C) TIF1γ mRNA and protein levels in A549 and H226 cells transfected with si-circPTK2 or si-NC. (D) siRNA-transfected A549 and H226 cells were serum-starved for 24 h and then treated with or without TGF-β1 (5 ng/ml) for 24 h and 48 h, respectively. Snail and N-cadherin protein levels were determined by western blot. (E, F) A549 and H226 cells were treated as above and subjected to the transwell migration and invasion assays. Migrated and invasive cells were stained and counted in at least three light microscopic fields. Scale bar, 100 μm. *P < 0.05; **P < 0.01; ***P < 0.001. (TIF 4550 kb)

Additional file 9: **Figure S7.** CircPTK2 overexpression attenuates NSCLC cell metastasis in vivo by the bioluminescent imaging. (A) CircPTK2 expression in A549 cells stably overexpressing circPTK2. pLV-Luci(2A)Puro lentiviral expression vector (*upper panel*) was used to stably overexpress circPTK2. The empty vector was served as negative control. CircPTK2 expression was determined by qRT-PCR (*bottom panel*). (B) Schematic flowchart of the in vivo metastasis experiments with A549 cells stably transfected with pLV-circPTK2 or vector (i.v.) and TGF-β1 (i.p.) injected into BALB/c nude mice (*n* = 10 mice per group in circPTK2 + TGF-β1 and vector + TGF-β1). (C) Representative images of in vivo bioluminescence of mice injected with circPTK2-overexpressed A549 cells or vector-control cells at day 42 post-inoculation. Color bar represents extent of luciferase bioluminescence intensity (blue, green and red indicate low, medium and high intensity, respectively). (D) Quantification of radiance emitted from active luciferase in lung of mice (*n* = 10 mice for each group) at day 42. (E) Representative images showing metastatic nodules established in lung taken from the mice injected with circPTK2-overexpressed A549 cells or vector control cells at day 49 (*upper*). Scale bar, 4 mm. H&E staining was performed for histological confirmation of metastasizing tumor cells in lung (*bottom*). Scale bar, 100 μm. Green arrowhead indicate micrometastasis. (F) Gross view of liver of mice at day 49 post-inoculation (*upper*) and microscopic images of H&E staining for liver metastases (*bottom*). Scale bar, 4 mm or 100 μm. Green arrowhead indicate micrometastasis. (G) Dot plots showing the distribution of the number of micrometastases in per section of liver (*n* = 10 mice for each group). *P < 0.05; **P < 0.01; ***P < 0.001. (TIF 5869 kb)

Additional file 10: **Table S3.** Demographic and clinical characteristics of 73 NSCLC patients and relative expression of circPTK2 and TIF1γ mRNA in 73 paired NSCLC tissues. (DOC 134 kb)

Additional file 11: **Figure S8.** A work model of the mechanistic interaction between circPTK2, miR-429/miR-200b-3p and TIF1γ for controlling TGF-β-induced EMT in NSCLC cells: Circular RNA circPTK2 upregulates the expression of TIF1γ, a well-known negative regulator of TGF-β signaling, by sponging miR-429/miR-200b-3p in NSCLC cells, and in turn inhibits TGF-β-induced EMT. (TIF 449 kb)

Additional file 12: **Table S4.** Demographic and clinical characteristics of 73 NSCLC patients and relative expression of miR-429 and miR-200b-3p in 73 paired NSCLC tissues. (DOC 151 kb)

Additional file 13: **Figure S9.** miR-429/miR-200b-3p are upregulated in NSCLC tissues. (A, B) qRT-PCR analysis of miR-429/miR-200b-3p levels in 73 human NSCLC tumors and paired noncancerous lung tissues. (C, D) Relative expression of miR-429/miR-200b-3p in 73 paired NSCLC tissues. *Y*-axis represents the log_10_ transformed fold change of T/N expression ratios of miR-429 or miR-200b-3p. The number of each specimen is shown below *x*-axis. (E, F) Relative miR-429/miR-200b expression levels of 56 human NSCLC tumors and paired adjacent normal lung tissues in a public data set (GSE36681). Mean values are indicted by solid bars, and values are expressed as mean ± SEM. **P < 0.01; ***P < 0.001. (TIF 2543 kb)

Additional file 14: **Figure S10.** Expression of ZEB1/ZEB2 and miR-429/miR-200b-3p in A549 cells treated with TGF-β1 in time-dependent manner. After being serum-starved for 24 h, A549 cells were exposed to 5 ng/ml TGF-β1 for the indicated times, and the expression of ZEB1/ZEB2 (A, B) and miR-429/miR-200b-3p (C, D) were determined by qRT-PCR analysis. (TIF 2073 kb)

Additional file 15: **Figure S11.** TGF-β inhibits QKI expression and QKI knockdown reduces circPTK2 expression in A549 cells. (A, B) After being serum-starved for 24 h, A549 cells were exposed to 5 ng/ml TGF-β1 for the indicated times, and the expression of QKI and circPTK2 was determined by qRT-PCR analysis. (C, D) qRT-PCR analysis of QKI and circPTK2 levels in A549 cells transfected with two siRNAs specific for QKI (si-QKI-1 and si-QKI-2). Scramble siRNA was used as negative control (si-NC). **P < 0.01; ***P < 0.001. (TIF 557 kb)

Additional file 16: **Table S5.** Primers for qRT-PCR analysis. (DOC 31 kb)

## Abbreviations

CircRNA: Circular RNA
EMT: Epithelial-mesenchymal transition
FISH: Fluorescence in situ hybridization
miRNA: microRNA
NSCLC: Non-small cell lung cancer
PTK2: Protein tyrosine kinase 2
QKI: Quaking
RIP: RNA-binding protein immunoprecipitation
TGF-β: Transforming growth factor β
TIF1γ: Transcriptional intermediary factor 1γ
ZEB: Zinc finger E-box binding homeobox
