# Supplementary figures and images for: Circular RNA hsa_circ_0008305 (circPTK2) inhibits TGF-β-induced epithelial-mesenchymal transition and metastasis by controlling TIF1γ in non-small cell lung cancer
Source: Mol Cancer. 2018 Sep 27;17:140. doi: 10.1186/s12943-018-0889-7 (PMC6161470; doi:10.1186/s12943-018-0889-7)

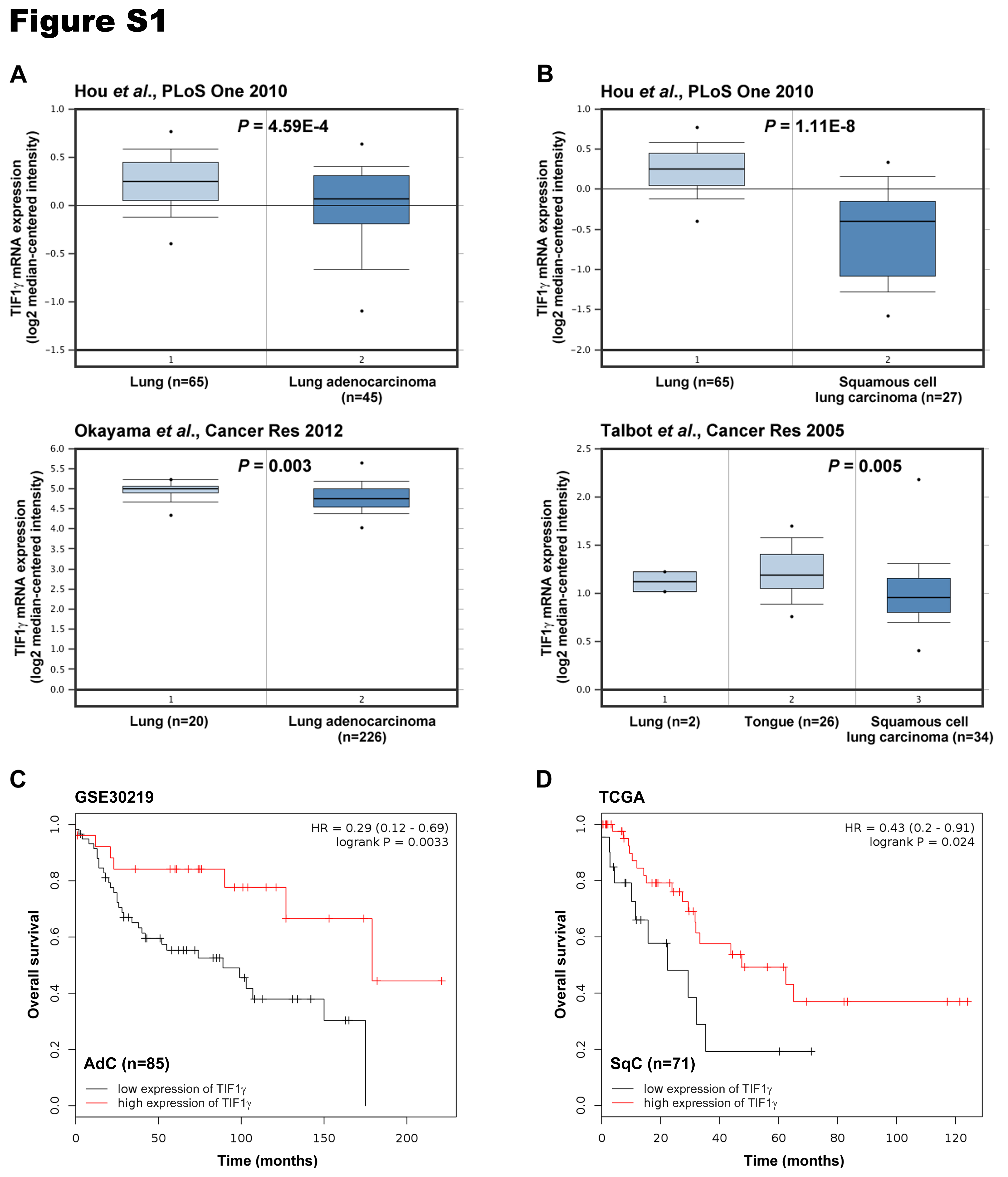

Supplement: Supplementary file 1 — Figure S1. Reduced TIF1γ is expressed in NSCLC tissues and associated with poor survival of NSCLC patients. (A, B) Data regarding TIF1γ mRNA expression in lung adenocarcinoma, squamous cell carcinoma tissues and normal lung tissues from several study groups in Oncomine database (http://www.oncomine.org). (C, D) Kaplan-Meier survival curves for 85 patients with lung adenocarcinoma (AdC) and 71 patients with lung squamous cell carcinoma (SqC). Primary data were taken from GSE30219 and TCGA in Kaplan-Meier Plotter (http://www.kmplot.com). (TIF 2402 kb) [file 12943_2018_889_MOESM1_ESM.tif]

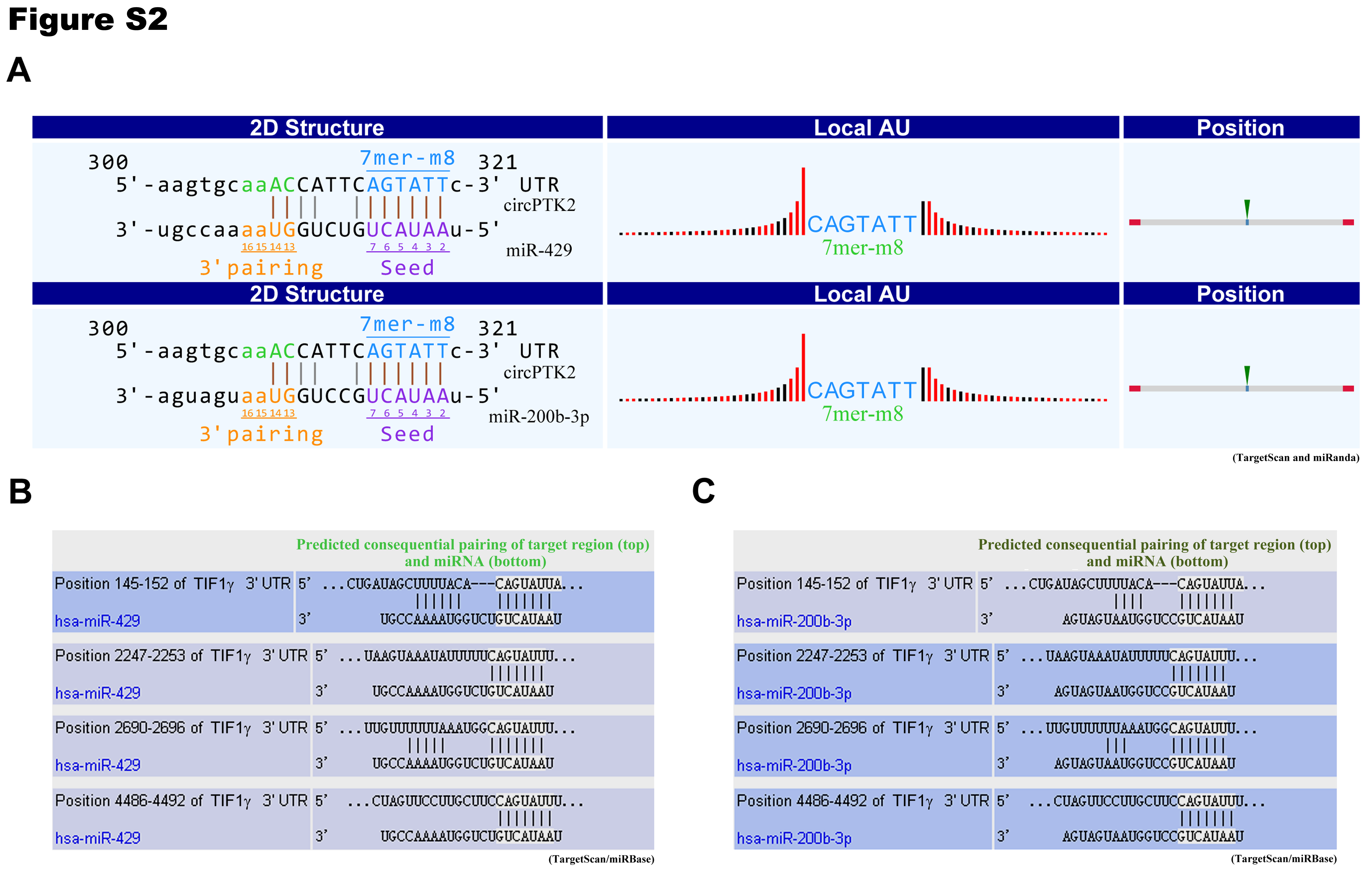

Supplement: Supplementary file 3 — Figure S2. The in silico prediction of the interaction between miR-429/miR-200b-3p and circPTK2 or TIF1γ 3’-UTR. (A) The interaction of circPTK2 and miR-429/miR-200b-3p was predicted with miRNA target prediction software (Arraystar’s home-made) based on TargetScan and miRanda. (B, C) The target interaction between miR-429/miR-200b-3p and TIF1γ 3’-UTR was in silico predicted by TargetScan (Release 7.1 http://www.targetscan.org)/miRBase (Release 21, http://www.mirbase.org). Four different sites (positions 145–152, 2247–2253, 2690–2696 and 4486–4492) of TIF1γ 3’-UTR were predicted to be targets of miR-429/miR-200b-3p. (TIF 6394 kb) [file 12943_2018_889_MOESM3_ESM.tif]

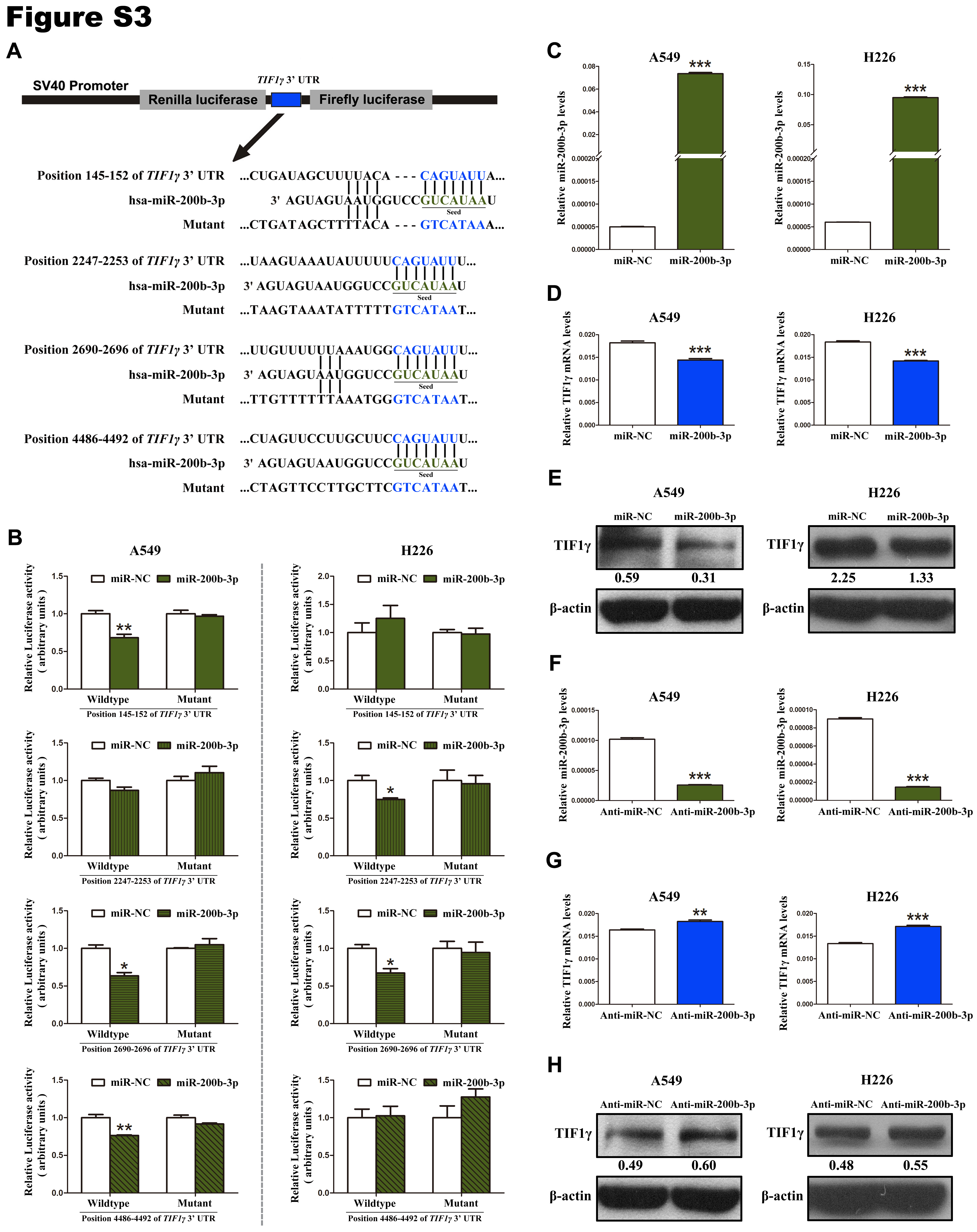

Supplement: Supplementary file 5 — Figure S3. miR-200b-3p inhibits TIF1γ expression by targeting 3’-UTR of TIF1γ transcript. (A) Schematic description for the subcloning of the predicted miR-200b-3p binding sites of TIF1γ 3’-UTR in psiCHECK-2 luciferase vector. Predicted duplex formation between miR-200b-3p and the wild-type/mutant of miR-200b-3p binding sites was indicated. The entire subcloning sequences were listed in Additional file 4: Table S2. (B) Relative luciferase activity of the wild-type/mutant TIF1γ 3’-UTR reporter gene in A549 and H226 cells transfected with miR-200b-3p or negative control (miR-NC). Scrambled sequence was used as miR-NC. Relative Renilla luciferase activity was determined after normalizing against the firefly luciferase activity. (C) qRT-PCR analysis of miR-200b-3p expression levels in A549 and H226 cells transfected with miR-200b-3p mimics or miR-NC. U6 was employed as internal control. (D, E) TIF1γ mRNA and protein expression in A549 and H226 cells transfected with miR-200b-3p mimics or miR-NC. β-actin was used as internal control. Densitometry values for TIF1γ protein were normalized to β-actin and shown below the corresponding bands. (F) miR-200b-3p expression levels in A549 and H226 cells transfected with miR-200b-3p inhibitors (anti-miR-200b-3p) or negative control (anti-miR-NC). Scrambled sequence was used as anti-miR-NC. (G, H) TIF1γ mRNA and protein expression in A549 and H226 cells transfected with anti-miR-200b-3p or anti-miR-NC. *P < 0.05; **P < 0.01; ***P < 0.001. (TIF 5059 kb) [file 12943_2018_889_MOESM5_ESM.tif]

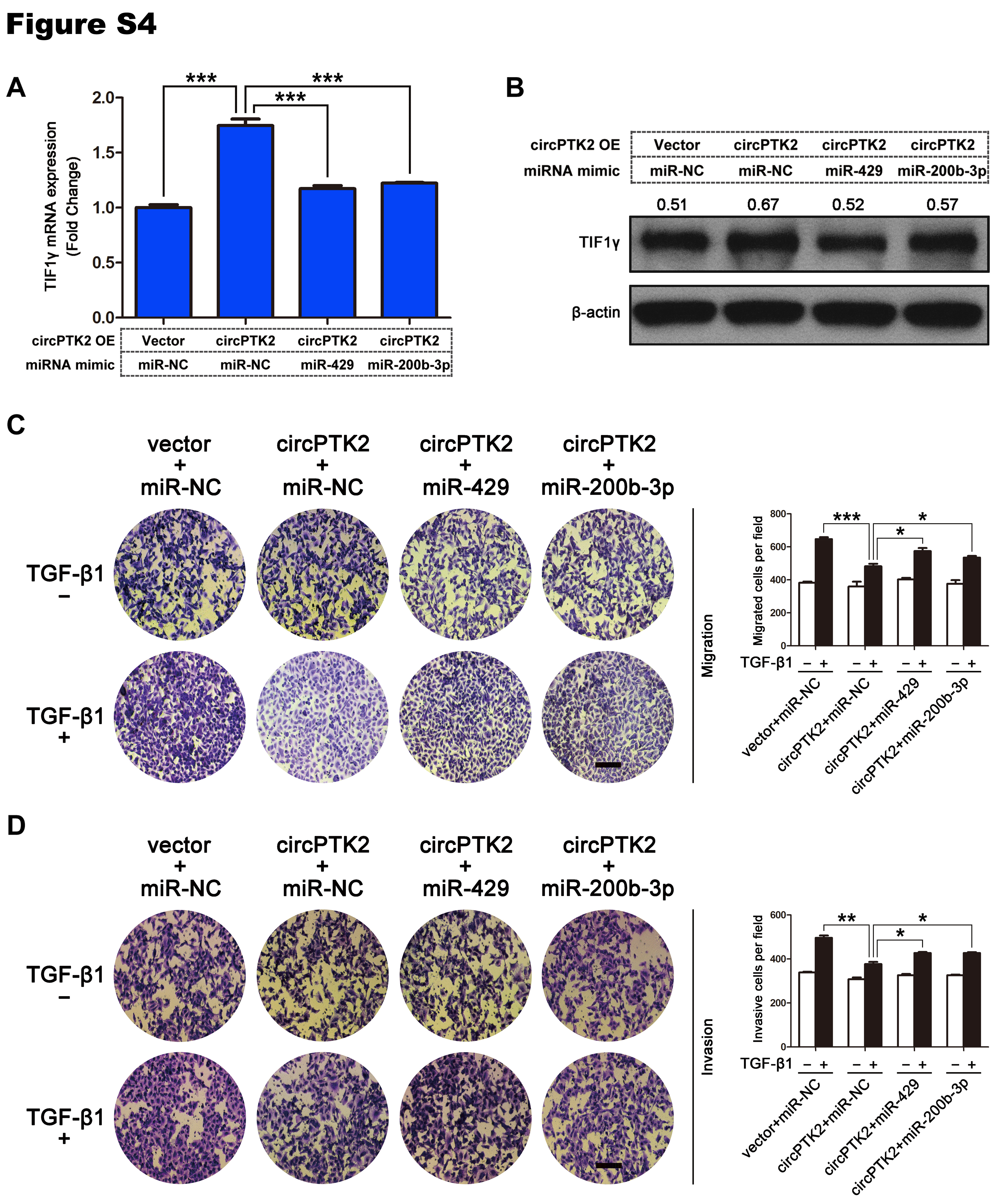

Supplement: Supplementary file 6 — Figure S4. CircPTK2 abolishes endogenous miR-429/miR-200b-3p-mediated repression of TIF1γ and inhibits TGF-β-induced invasion of NSCLC cells. (A) TIF1γ mRNA expression in A549 cells transiently overexpressing circPTK2 or empty vector in the presence or absence of miR-429/miR-200b-3p mimics. OE, overexpression. (B) TIF1γ protein levels in A549 cells transiently overexpressing circPTK2 in the above-mentioned condition. Densitometry values for TIF1γ protein were normalized to β-actin and indicated below the corresponding bands. (C, D) A549 cells overexpressing circPTK2 and miR-429/miR-200b-3p mimics were serum-starved for 24 h, and then were subjected to Transwell migration and invasion assays in the presence or absence of TGF-β1 described as Methods. Migrated and invasive cells were stained and counted in at least three light microscopic fields. Scale bar, 100 μm. *P < 0.05; **P < 0.01; ***P < 0.001. (TIF 14297 kb) [file 12943_2018_889_MOESM6_ESM.tif]

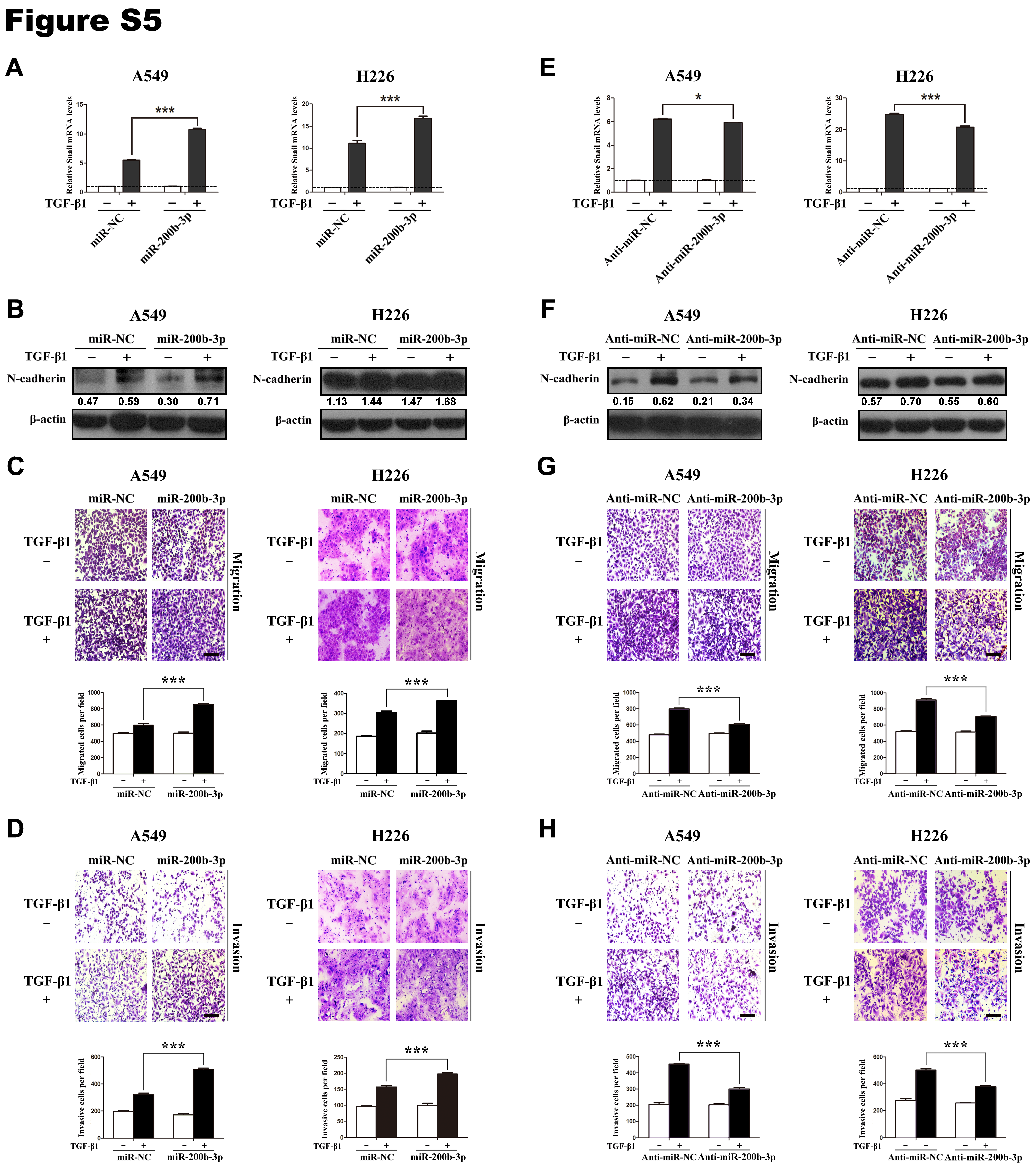

Supplement: Supplementary file 7 — Figure S5. miR-200b-3p promotes TGF-β-induced EMT and invasion in NSCLC cells. (A) After being serum-starved for 24 h, A549 and H226 cells transiently overexpressing miR-200b-3p were treated with or without TGF-β1 (5 ng/ml) for 1 h and 2 h, respectively. Snail mRNA expression was quantified by qRT-PCR analysis. Snail mRNA level of the unstimulated cells was assigned the value 1, and the relative Snail mRNA expression in TGF-β1-stimulated cells was recalculated accordingly. (B) After being serum-starved for 24 h, A549 and H226 cells transiently overexpressing miR-200b-3p were treated with or without TGF-β1 (5 ng/ml) for 24 h and 48 h, respectively. Western blot analysis was performed to examine the expression of N-cadherin, which was normalized to β-actin. (C) A549 and H226 cells transiently overexpressing miR-200b-3p were treated as above and allowed to migrate through an 8-μM pore in transwells. Migrated cells were stained and counted in at least three light microscopic fields. Scale bar, 100 μm. (D) Cells were treated as above and allowed to invade through Matrigel-coated membrane in transwells. Invasive cells were stained and counted under a light microscope. Scale bar, 100 μm. (E) After being serum-starved for 24 h, A549 and H226 cells transiently overexpressing anti-miR-200b-3p were treated with or without TGF-β1 (5 ng/ml) for 1 h and 2 h, respectively. qRT-PCR analysis was done to determine the relative Snail mRNA expression. (F) After being serum-starved for 24 h, A549 and H226 cells transiently overexpressing anti-miR-200b-3p were treated with or without TGF-β1 (5 ng/ml) for 24 h and 48 h, respectively. N-cadherin expression was analyzed by western blot. (G) A549 and H226 cells transiently overexpressing anti-miR-200b-3p were treated as above and allowed to migrate through an 8-μM pore in transwells. Migrated cells were stained and counted in at least three light microscopic fields. Scale bar, 100 μm. (H) Cells were treated as above and allowed to invade thr [file 12943_2018_889_MOESM7_ESM.tif]

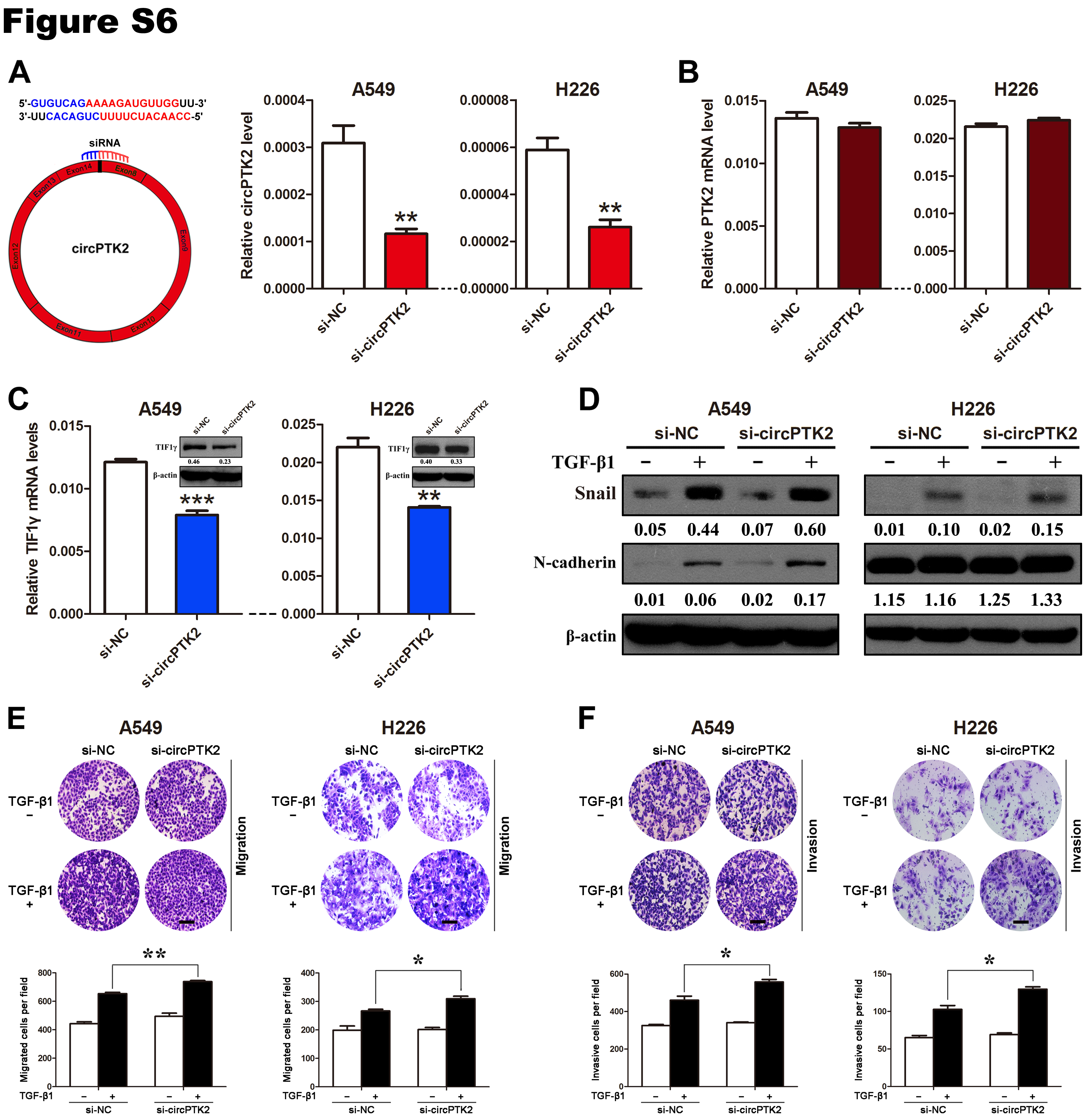

Supplement: Supplementary file 8 — Figure S6. CircPTK2 knockdown inhibits TIF1γ expression and promotes TGF-β-induced EMT and invasion of NSCLC cells in vitro. (A) Left panel, a siRNA targeting circPTK2 JCT (si-circPTK2) was designed to specifically knockdown circPTK2. Right panel, A549 and H226 cells were transfected with si-circPTK2 and siRNA negative control (si-NC). qRT-PCR was performed to detect circPTK2 expression in the siRNA-transfected cells. (B) Linear PTK2 mRNA expression in circPTK2-silenced A549 and H226 cells. (C) TIF1γ mRNA and protein levels in A549 and H226 cells transfected with si-circPTK2 or si-NC. (D) siRNA-transfected A549 and H226 cells were serum-starved for 24 h and then treated with or without TGF-β1 (5 ng/ml) for 24 h and 48 h, respectively. Snail and N-cadherin protein levels were determined by western blot. (E, F) A549 and H226 cells were treated as above and subjected to the transwell migration and invasion assays. Migrated and invasive cells were stained and counted in at least three light microscopic fields. Scale bar, 100 μm. *P < 0.05; **P < 0.01; ***P < 0.001. (TIF 4550 kb) [file 12943_2018_889_MOESM8_ESM.tif]

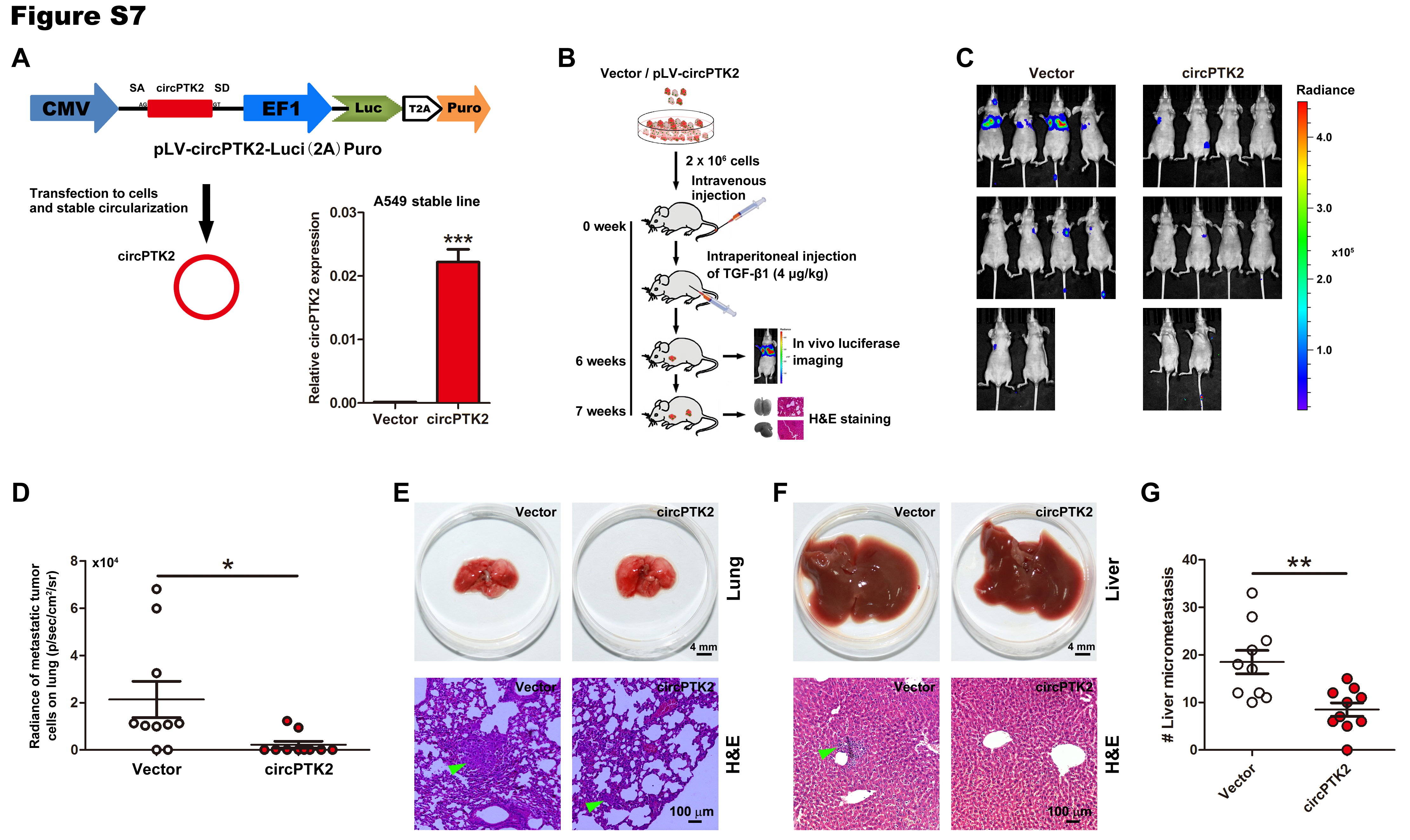

Supplement: Supplementary file 9 — Figure S7. CircPTK2 overexpression attenuates NSCLC cell metastasis in vivo by the bioluminescent imaging. (A) CircPTK2 expression in A549 cells stably overexpressing circPTK2. pLV-Luci(2A)Puro lentiviral expression vector (upper panel) was used to stably overexpress circPTK2. The empty vector was served as negative control. CircPTK2 expression was determined by qRT-PCR (bottom panel). (B) Schematic flowchart of the in vivo metastasis experiments with A549 cells stably transfected with pLV-circPTK2 or vector (i.v.) and TGF-β1 (i.p.) injected into BALB/c nude mice (n = 10 mice per group in circPTK2 + TGF-β1 and vector + TGF-β1). (C) Representative images of in vivo bioluminescence of mice injected with circPTK2-overexpressed A549 cells or vector-control cells at day 42 post-inoculation. Color bar represents extent of luciferase bioluminescence intensity (blue, green and red indicate low, medium and high intensity, respectively). (D) Quantification of radiance emitted from active luciferase in lung of mice (n = 10 mice for each group) at day 42. (E) Representative images showing metastatic nodules established in lung taken from the mice injected with circPTK2-overexpressed A549 cells or vector control cells at day 49 (upper). Scale bar, 4 mm. H&E staining was performed for histological confirmation of metastasizing tumor cells in lung (bottom). Scale bar, 100 μm. Green arrowhead indicate micrometastasis. (F) Gross view of liver of mice at day 49 post-inoculation (upper) and microscopic images of H&E staining for liver metastases (bottom). Scale bar, 4 mm or 100 μm. Green arrowhead indicate micrometastasis. (G) Dot plots showing the distribution of the number of micrometastases in per section of liver (n = 10 mice for each group). *P < 0.05; **P < 0.01; ***P < 0.001. (TIF 5869 kb) [file 12943_2018_889_MOESM9_ESM.tif]

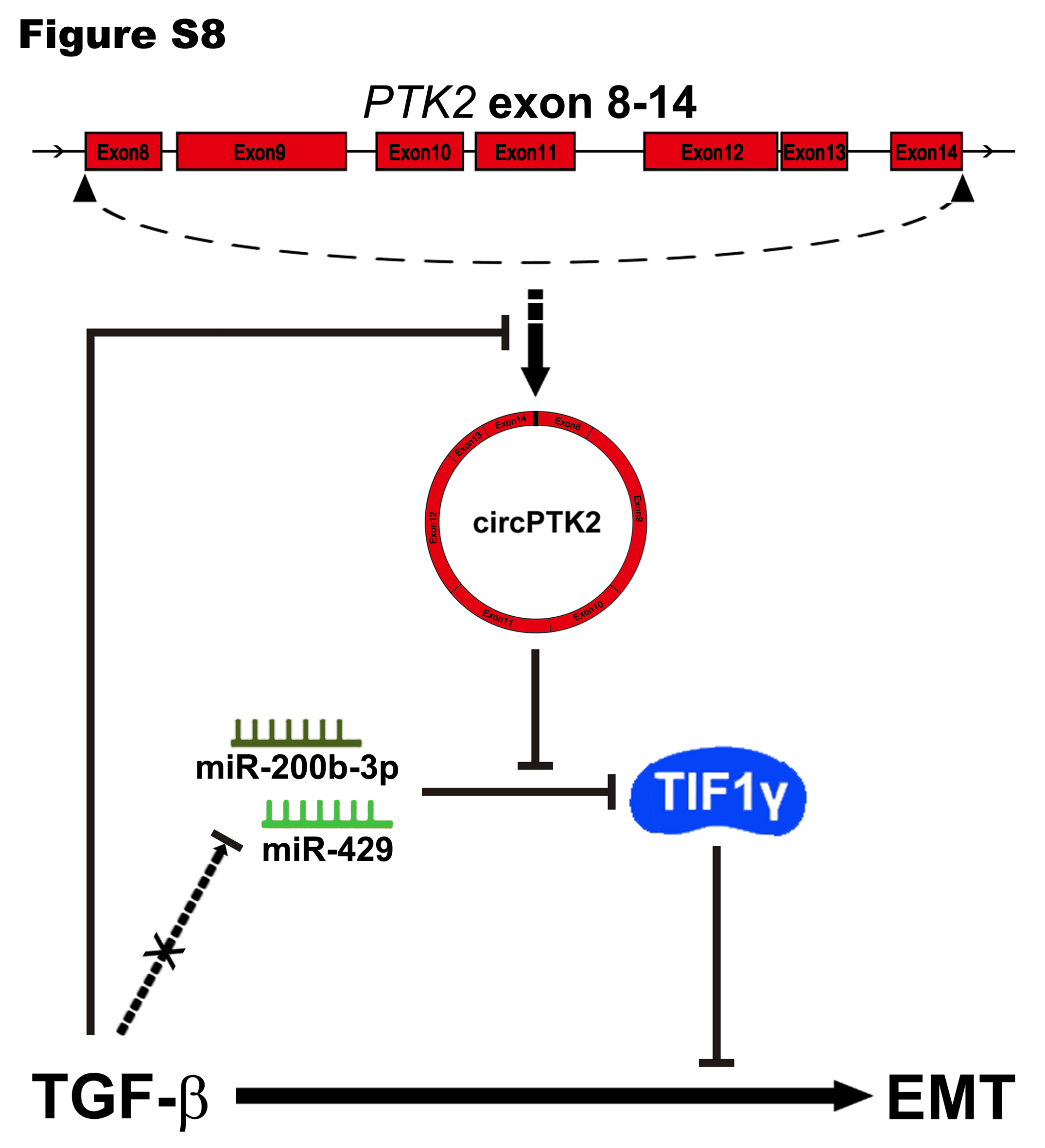

Supplement: Supplementary file 11 — Figure S8. A work model of the mechanistic interaction between circPTK2, miR-429/miR-200b-3p and TIF1γ for controlling TGF-β-induced EMT in NSCLC cells: Circular RNA circPTK2 upregulates the expression of TIF1γ, a well-known negative regulator of TGF-β signaling, by sponging miR-429/miR-200b-3p in NSCLC cells, and in turn inhibits TGF-β-induced EMT. (TIF 449 kb) [file 12943_2018_889_MOESM11_ESM.tif]

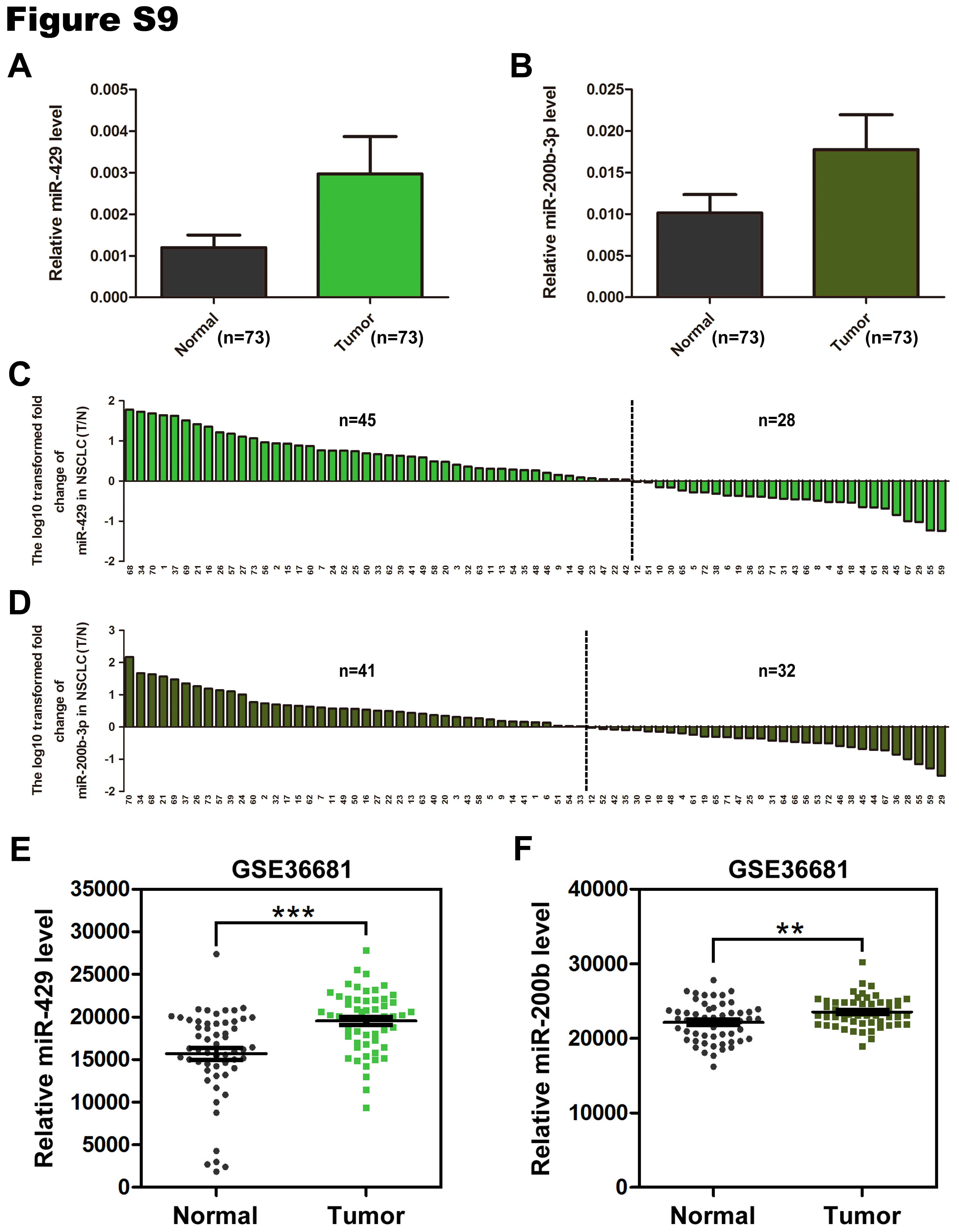

Supplement: Supplementary file 13 — Figure S9. miR-429/miR-200b-3p are upregulated in NSCLC tissues. (A, B) qRT-PCR analysis of miR-429/miR-200b-3p levels in 73 human NSCLC tumors and paired noncancerous lung tissues. (C, D) Relative expression of miR-429/miR-200b-3p in 73 paired NSCLC tissues. Y-axis represents the log10 transformed fold change of T/N expression ratios of miR-429 or miR-200b-3p. The number of each specimen is shown below x-axis. (E, F) Relative miR-429/miR-200b expression levels of 56 human NSCLC tumors and paired adjacent normal lung tissues in a public data set (GSE36681). Mean values are indicted by solid bars, and values are expressed as mean ± SEM. **P < 0.01; ***P < 0.001. (TIF 2543 kb) [file 12943_2018_889_MOESM13_ESM.tif]

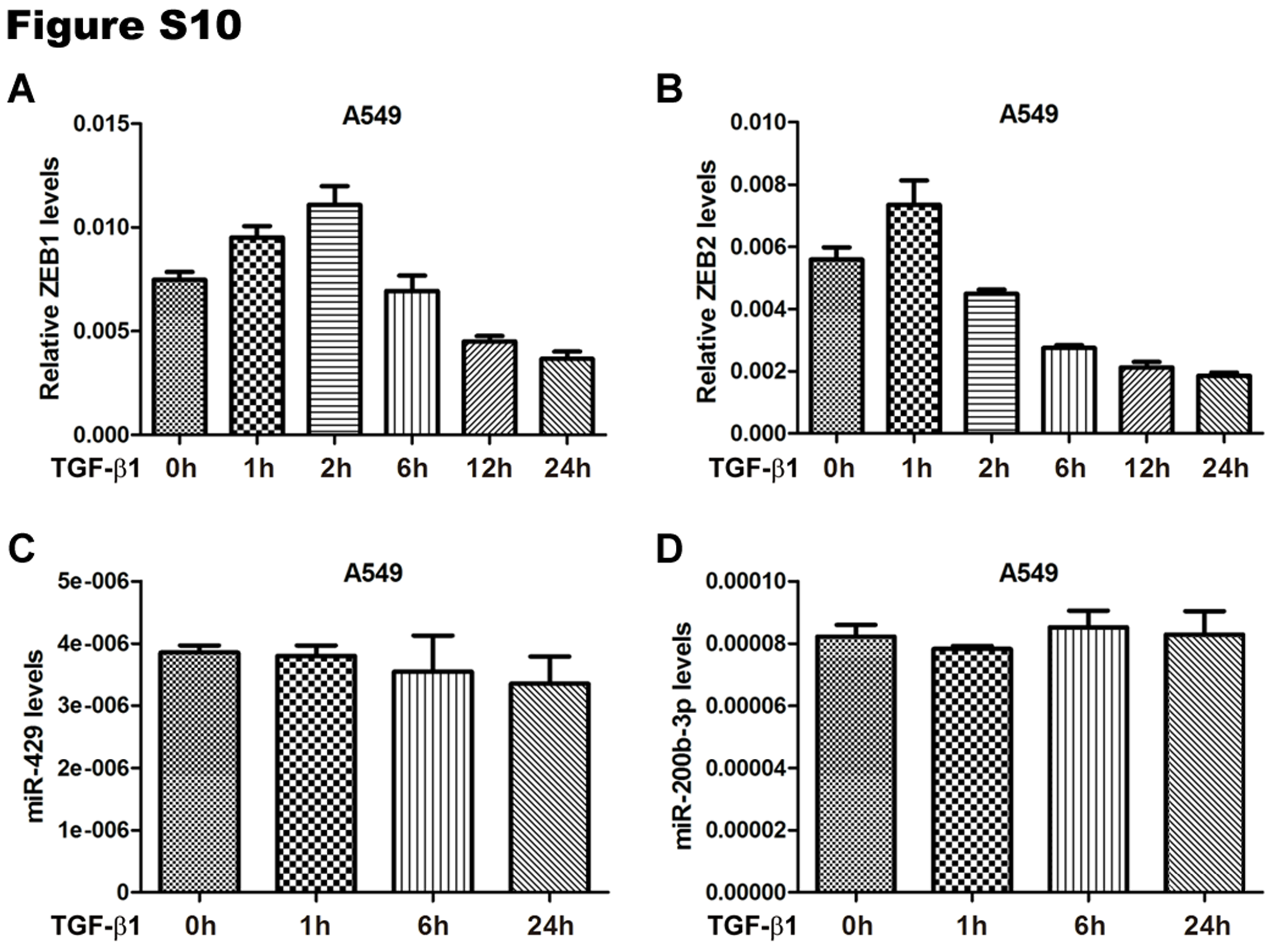

Supplement: Supplementary file 14 — Figure S10. Expression of ZEB1/ZEB2 and miR-429/miR-200b-3p in A549 cells treated with TGF-β1 in time-dependent manner. After being serum-starved for 24 h, A549 cells were exposed to 5 ng/ml TGF-β1 for the indicated times, and the expression of ZEB1/ZEB2 (A, B) and miR-429/miR-200b-3p (C, D) were determined by qRT-PCR analysis. (TIF 2073 kb) [file 12943_2018_889_MOESM14_ESM.tif]

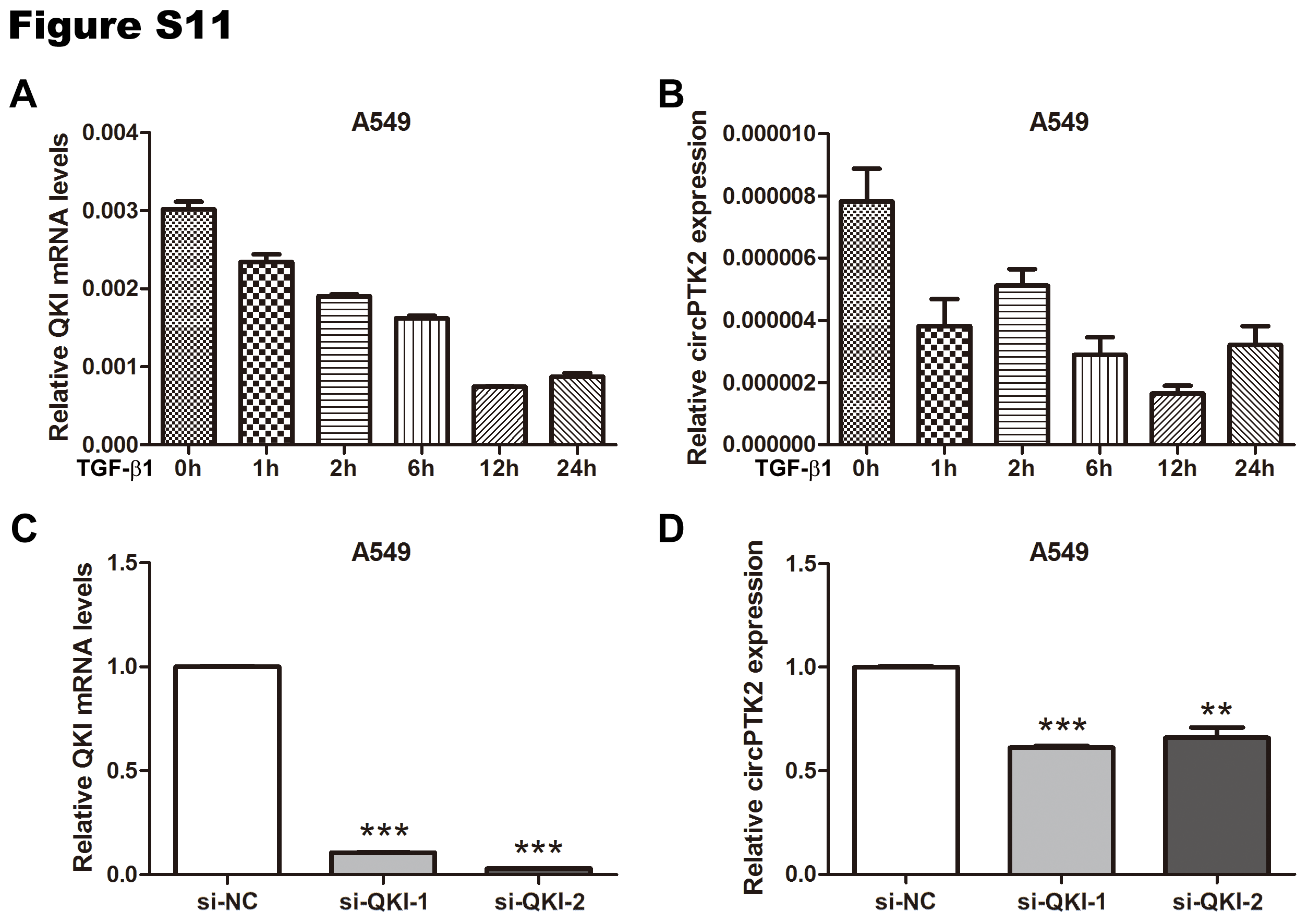

Supplement: Supplementary file 15 — Figure S11. TGF-β inhibits QKI expression and QKI knockdown reduces circPTK2 expression in A549 cells. (A, B) After being serum-starved for 24 h, A549 cells were exposed to 5 ng/ml TGF-β1 for the indicated times, and the expression of QKI and circPTK2 was determined by qRT-PCR analysis. (C, D) qRT-PCR analysis of QKI and circPTK2 levels in A549 cells transfected with two siRNAs specific for QKI (si-QKI-1 and si-QKI-2). Scramble siRNA was used as negative control (si-NC). **P < 0.01; ***P < 0.001. (TIF 557 kb) [file 12943_2018_889_MOESM15_ESM.tif]
