# Supplementary material for: Circular RNA hsa_circ_0008305 (circPTK2) inhibits TGF-β-induced epithelial-mesenchymal transition and metastasis by controlling TIF1γ in non-small cell lung cancer
Source: Mol Cancer. 2018 Sep 27;17:140. doi: 10.1186/s12943-018-0889-7 (PMC6161470; doi:10.1186/s12943-018-0889-7)
Supplement: Supplementary file 2 — Table S1. Arraystar Human circRNA Array analysis of A549 cells treated with TGF-β1. Among 187 differentially expressed circRNAs (fold change ≥1.5, P-value < 0.05 and FDR < 0.05), 88 circRNAs were up-regulated and 99 circRNAs were down-regulated in A549 cells after TGF-β1 treatment for 24 h. CircRNA ID: encoded in circBase (http://www.circbase.org). P-value: estimated by paired t-test. FDR: false discovery rate, calculated from Benjamini Hochberg FDR. Fold change: the absolute ratio (no log scale) of normalized intensities between two conditions (treated with TGF-β1 vs. treated without TGF-β1). (DOC 195 kb) [file 12943_2018_889_MOESM2_ESM.doc]

**Table S1. Arraystar Human circRNA Array analysis of A549 cells treated with TGF-β1.** Among 187 differentially expressed circRNAs (fold change ≥1.5, P-value <0.05 and FDR <0.05), 88 circRNAs were up-regulated and 99 circRNAs were down-regulated in A549 cells after TGF-β1 treatment for 24 h. CircRNA ID: encoded in circBase ([http://www.circbase.org](http://www.circbase.org/)). P-value: estimated by paired t-test. FDR: false discovery rate, calculated from Benjamini Hochberg FDR. Fold change: the absolute ratio (no log scale) of normalized intensities between two conditions (treated with TGF-β1 vs. treated without TGF-β1).

| **Up-regulated circRNAs (88)** | | | | | | **Down-regulated circRNAs (99)** | | | | | |
| --- | --- | --- | --- | --- | --- | --- | --- | --- | --- | --- | --- |
| **CircRNA ID** | **CircRNA type** | **Chr. position** | **P-value** | **FDR** | **Fold change** | **CircRNA ID** | **CircRNA type** | **Chr. position** | **P-value** | **FDR** | **Fold change** |
| hsa_circ_0061265 | exonic | chr21 | 5.52348E-05 | 0.001009478 | 3.8411282 | hsa_circ_0026218 | exonic | chr12 | 5.63886E-06 | 0.000418472 | 3.1771193 |
| hsa_circ_0072279 | exonic | chr5 | 4.97865E-06 | 0.000408763 | 2.4630932 | hsa_circ_0022603 | exonic | chr11 | 0.000349941 | 0.002866235 | 3.080726 |
| hsa_circ_0008035 | exonic | chr8 | 0.001921568 | 0.008571802 | 2.4388646 | hsa_circ_0035445 | exonic | chr15 | 3.2499E-07 | 0.00012463 | 2.9000624 |
| hsa_circ_0075410 | exonic | chr6 | 4.57013E-05 | 0.000929676 | 2.3668694 | hsa_circ_0078297 | exonic | chr6 | 4.66497E-05 | 0.000929676 | 2.7059957 |
| hsa_circ_0060927 | exonic | chr20 | 9.36774E-05 | 0.001333813 | 2.3087664 | hsa_circ_0059955 | exonic | chr20 | 7.97879E-06 | 0.000486561 | 2.6832155 |
| hsa_circ_0087888 | exonic | chr9 | 3.5623E-07 | 0.00012463 | 2.2122934 | hsa_circ_0000720 | exonic | chr16 | 1.56881E-05 | 0.000567256 | 2.6826082 |
| hsa_circ_0002714 | exonic | chr7 | 0.000183014 | 0.00192361 | 2.1842326 | hsa_circ_0046188 | exonic | chr17 | 1.74688E-05 | 0.000567256 | 2.505895 |
| hsa_circ_0087643 | exonic | chr9 | 0.006625068 | 0.02098938 | 2.1814828 | hsa_circ_0003915 | exonic | chr2 | 6.15076E-05 | 0.00104802 | 2.4333768 |
| hsa_circ_0060937 | exonic | chr20 | 0.000229301 | 0.002246231 | 2.1236229 | hsa_circ_0004463 | exonic | chr1 | 3.52993E-05 | 0.000778768 | 2.3920456 |
| hsa_circ_0076058 | exonic | chr6 | 5.16032E-05 | 0.000972125 | 2.0106336 | hsa_circ_0007422 | exonic | chr2 | 8.72354E-06 | 0.000486561 | 2.3875421 |
| hsa_circ_0002456 | exonic | chr10 | 7.0152E-07 | 0.000171802 | 1.9566016 | hsa_circ_0000965 | intragenic | chr19 | 3.53255E-05 | 0.000778768 | 2.3610573 |
| hsa_circ_0004365 | exonic | chr7 | 0.000300314 | 0.002642505 | 1.9561812 | hsa_circ_0000288 | exonic | chr11 | 2.99541E-05 | 0.000733668 | 2.3496798 |
| hsa_circ_0018814 | exonic | chr10 | 0.000108135 | 0.001419534 | 1.9555004 | hsa_circ_0000091 | exonic | chr1 | 6.02846E-05 | 0.00104707 | 2.3247648 |
| hsa_circ_0002733 | exonic | chr1 | 4.21343E-06 | 0.000408763 | 1.9262584 | hsa_circ_0079480 | exonic | chr7 | 0.000920048 | 0.005261062 | 2.3106097 |
| hsa_circ_0006055 | exonic | chr20 | 2.57705E-05 | 0.00067345 | 1.8987081 | hsa_circ_0002437 | exonic | chr1 | 0.000375986 | 0.002956214 | 2.1431903 |
| hsa_circ_0044556 | exonic | chr17 | 2.53298E-05 | 0.00067345 | 1.8768591 | hsa_circ_0068462 | exonic | chr3 | 2.38358E-05 | 0.00065197 | 2.1282155 |
| hsa_circ_0032821 | exonic | chr14 | 0.004550322 | 0.015919627 | 1.8667376 | hsa_circ_0044436 | exonic | chr17 | 1.11416E-06 | 0.000227381 | 2.1167968 |
| hsa_circ_0000144 | antisense | chr1 | 0.003227477 | 0.012376292 | 1.8584744 | hsa_circ_0072386 | exonic | chr5 | 4.69373E-05 | 0.000929676 | 2.0556947 |
| hsa_circ_0005571 | exonic | chr19 | 1.46868E-05 | 0.000567256 | 1.8468727 | hsa_circ_0004519 | exonic | chr16 | 7.63617E-05 | 0.001191145 | 2.0541077 |
| hsa_circ_0001295 | exonic | chr3 | 0.000494947 | 0.00345335 | 1.8389239 | hsa_circ_0013912 | exonic | chr1 | 9.39069E-06 | 0.000486561 | 2.0333808 |
| hsa_circ_0076055 | exonic | chr6 | 0.000795135 | 0.00470359 | 1.7767913 | hsa_circ_0006446 | exonic | chr19 | 7.87974E-06 | 0.000486561 | 2.0125084 |
| hsa_circ_0070396 | exonic | chr4 | 0.004868623 | 0.016817006 | 1.7762889 | hsa_circ_0003162 | exonic | chr7 | 0.000299738 | 0.002642505 | 1.9726333 |
| hsa_circ_0005038 | exonic | chr6 | 4.06254E-05 | 0.000865144 | 1.7753475 | hsa_circ_0029634 | exonic | chr13 | 2.74591E-05 | 0.000700494 | 1.9578051 |
| hsa_circ_0084151 | exonic | chr8 | 4.71577E-06 | 0.000408763 | 1.7602627 | hsa_circ_0007551 | exonic | chr5 | 0.001181422 | 0.006116919 | 1.9296981 |
| hsa_circ_0067716 | exonic | chr3 | 8.03833E-05 | 0.001238105 | 1.7597697 | hsa_circ_0004099 | exonic | chr11 | 2.5671E-07 | 0.00012463 | 1.9071021 |
| hsa_circ_0002675 | exonic | chr9 | 0.001057876 | 0.005731724 | 1.7371783 | hsa_circ_0000247 | exonic | chr10 | 9.9405E-07 | 0.000221312 | 1.8900055 |
| hsa_circ_0012576 | exonic | chr1 | 0.000400138 | 0.003030504 | 1.7326997 | hsa_circ_0072088 | exonic | chr5 | 0.007127477 | 0.022179405 | 1.8451117 |
| hsa_circ_0013587 | exonic | chr1 | 0.000371013 | 0.002947451 | 1.7264363 | hsa_circ_0000977 | exonic | chr2 | 3.1059E-05 | 0.00074572 | 1.8431641 |
| hsa_circ_0008261 | exonic | chr2 | 1.22832E-05 | 0.000546938 | 1.7164461 | hsa_circ_0082306 | exonic | chr7 | 0.018144506 | 0.045204369 | 1.8371355 |
| hsa_circ_0088485 | exonic | chr9 | 2.84903E-05 | 0.000719307 | 1.7158696 | hsa_circ_0000822 | antisense | chr18 | 1.7366E-05 | 0.000567256 | 1.8342283 |
| hsa_circ_0092332 | intronic | chr19 | 0.001139512 | 0.00600143 | 1.7135917 | hsa_circ_0062760 | exonic | chr22 | 0.005524834 | 0.018376739 | 1.8192681 |
| hsa_circ_0016404 | exonic | chr1 | 0.000396913 | 0.003030504 | 1.7133099 | hsa_circ_0052318 | exonic | chr19 | 4.87679E-05 | 0.000949261 | 1.818872 |
| hsa_circ_0006913 | exonic | chr1 | 9.07816E-06 | 0.000486561 | 1.7118724 | hsa_circ_0007767 | exonic | chr11 | 5.26773E-06 | 0.000408763 | 1.8187306 |
| hsa_circ_0004712 | exonic | chr6 | 0.003205266 | 0.012322915 | 1.7115682 | hsa_circ_0083172 | exonic | chr7 | 0.000111949 | 0.001424915 | 1.8150934 |
| hsa_circ_0091894 | exonic | chrX | 0.000974426 | 0.005399025 | 1.7060973 | hsa_circ_0005556 | exonic | chr2 | 0.000258413 | 0.00240645 | 1.8094657 |
| hsa_circ_0004565 | exonic | chr3 | 9.20309E-05 | 0.001333813 | 1.7015388 | hsa_circ_0003600 | exonic | chr1 | 3.26242E-06 | 0.00038046 | 1.8069601 |
| hsa_circ_0020313 | exonic | chr10 | 0.000469114 | 0.003301322 | 1.6835616 | hsa_circ_0006220 | exonic | chr17 | 3.18657E-06 | 0.00038046 | 1.806812 |
| hsa_circ_0000700 | intronic | chr16 | 8.70624E-06 | 0.000486561 | 1.6770517 | hsa_circ_0000253 | intronic | chr10 | 1.03957E-05 | 0.000498649 | 1.8057595 |
| hsa_circ_0003502 | exonic | chr3 | 1.3693E-07 | 0.00012463 | 1.671348 | hsa_circ_0083294 | exonic | chr8 | 0.001968866 | 0.008690494 | 1.7874523 |
| hsa_circ_0007608 | exonic | chr1 | 1.5134E-06 | 0.000265314 | 1.6624788 | hsa_circ_0002696 | exonic | chr16 | 0.00027621 | 0.002539296 | 1.7859335 |
| hsa_circ_0056439 | exonic | chr2 | 9.33657E-05 | 0.001333813 | 1.6600534 | hsa_circ_0032253 | exonic | chr14 | 9.48535E-06 | 0.000486561 | 1.7859122 |
| hsa_circ_0008719 | exonic | chr19 | 2.2461E-07 | 0.00012463 | 1.6554751 | hsa_circ_0001568 | intronic | chr6 | 6.98719E-06 | 0.000486561 | 1.782425 |
| hsa_circ_0004006 | exonic | chr17 | 0.000171639 | 0.001868197 | 1.652642 | hsa_circ_0067772 | exonic | chr3 | 0.004076315 | 0.014594875 | 1.7605424 |
| hsa_circ_0009135 | exonic | chr1 | 4.88391E-05 | 0.000949261 | 1.6524163 | hsa_circ_0001296 | exonic | chr3 | 5.13577E-06 | 0.000408763 | 1.7526282 |
| hsa_circ_0000961 | intronic | chr19 | 0.000120973 | 0.001446789 | 1.633323 | hsa_circ_0007888 | exonic | chr20 | 3.36898E-05 | 0.000771088 | 1.7447904 |
| hsa_circ_0078768 | exonic | chr6 | 7.97526E-06 | 0.000486561 | 1.6289967 | hsa_circ_0043947 | exonic | chr17 | 0.000280709 | 0.002565135 | 1.733033 |
| hsa_circ_0000661 | antisense | chr15 | 4.66373E-06 | 0.000408763 | 1.6275121 | hsa_circ_0044638 | exonic | chr17 | 0.000378443 | 0.002956214 | 1.7316084 |
| hsa_circ_0006901 | exonic | chr4 | 0.00012579 | 0.001489902 | 1.6268974 | hsa_circ_0065898 | exonic | chr3 | 6.4714E-07 | 0.000171802 | 1.7300184 |
| hsa_circ_0024609 | exonic | chr11 | 8.13931E-06 | 0.000486561 | 1.6238255 | hsa_circ_0043278 | exonic | chr17 | 1.5167E-06 | 0.000265314 | 1.7250442 |
| hsa_circ_0045006 | exonic | chr17 | 0.000140226 | 0.001604739 | 1.6217117 | hsa_circ_0049888 | exonic | chr19 | 0.000338473 | 0.002809896 | 1.7213846 |
| hsa_circ_0001346 | exonic | chr3 | 6.16231E-05 | 0.00104802 | 1.6208555 | hsa_circ_0092328 | intronic | chr9 | 0.004799208 | 0.016671291 | 1.7161555 |
| hsa_circ_0044695 | exonic | chr17 | 0.000167188 | 0.001827874 | 1.6199541 | hsa_circ_0007874 | exonic | chr6 | 0.006281647 | 0.020241781 | 1.7083526 |
| hsa_circ_0005889 | exonic | chr15 | 0.001165541 | 0.006084007 | 1.6168428 | hsa_circ_0060733 | exonic | chr20 | 6.12605E-05 | 0.00104802 | 1.7070894 |
| hsa_circ_0060558 | exonic | chr20 | 0.000215714 | 0.002165092 | 1.6155351 | hsa_circ_0028173 | exonic | chr12 | 3.62342E-05 | 0.000785287 | 1.7047264 |
| hsa_circ_0000436 | intronic | chr12 | 0.000602905 | 0.003865219 | 1.6150801 | hsa_circ_0000305 | intronic | chr11 | 0.000285841 | 0.002582272 | 1.7012262 |
| hsa_circ_0000919 | intronic | chr19 | 0.000365876 | 0.00292949 | 1.6099754 | hsa_circ_0075504 | exonic | chr6 | 0.000648065 | 0.004011977 | 1.700948 |
| hsa_circ_0060848 | exonic | chr20 | 0.000418575 | 0.003087621 | 1.6071087 | hsa_circ_0000714 | exonic | chr16 | 0.000591784 | 0.00383407 | 1.6970372 |
| hsa_circ_0091934 | exonic | chrX | 0.003952308 | 0.014192378 | 1.6021779 | hsa_circ_0001644 | intronic | chr6 | 0.002468318 | 0.010041378 | 1.682862 |
| hsa_circ_0010501 | exonic | chr1 | 0.000178404 | 0.001907912 | 1.5929677 | hsa_circ_0092342 | intronic | chr11 | 0.018614309 | 0.04615204 | 1.6742897 |
| hsa_circ_0001745 | antisense | chr7 | 1.88218E-05 | 0.000568605 | 1.5877765 | hsa_circ_0004354 | exonic | chr16 | 0.000636821 | 0.003978506 | 1.6674935 |
| hsa_circ_0034072 | exonic | chr15 | 9.28795E-05 | 0.001333813 | 1.5840407 | hsa_circ_0052877 | exonic | chr2 | 3.25677E-06 | 0.00038046 | 1.6670619 |
| hsa_circ_0044413 | exonic | chr17 | 0.001244329 | 0.006309239 | 1.5818976 | hsa_circ_0005918 | exonic | chr11 | 0.000106001 | 0.001415527 | 1.6650642 |
| hsa_circ_0002818 | exonic | chrX | 0.000115792 | 0.001446789 | 1.5787266 | hsa_circ_0035381 | exonic | chr15 | 1.40453E-05 | 0.000567256 | 1.6641917 |
| hsa_circ_0044396 | exonic | chr17 | 0.000126952 | 0.001494495 | 1.5783648 | hsa_circ_0029605 | exonic | chr13 | 0.015352884 | 0.03989064 | 1.6617794 |
| hsa_circ_0088807 | exonic | chr9 | 0.000571496 | 0.003775857 | 1.576746 | hsa_circ_0005397 | exonic | chr17 | 0.000175487 | 0.00189325 | 1.6612794 |
| hsa_circ_0055548 | exonic | chr2 | 0.001456199 | 0.007089923 | 1.5712846 | hsa_circ_0084984 | exonic | chr8 | 0.000111901 | 0.001424915 | 1.6409928 |
| hsa_circ_0063331 | exonic | chr22 | 9.06655E-05 | 0.001329579 | 1.564159 | hsa_circ_0004066 | exonic | chr19 | 0.000613868 | 0.003904835 | 1.633539 |
| hsa_circ_0036629 | exonic | chr15 | 9.4479E-05 | 0.001337451 | 1.5623956 | hsa_circ_0020390 | exonic | chr10 | 0.000487409 | 0.003410473 | 1.6217234 |
| hsa_circ_0092290 | intronic | chr8 | 0.000120251 | 0.001446789 | 1.5547801 | hsa_circ_0001666 | exonic | chr6 | 0.000181393 | 0.001916184 | 1.6199031 |
| hsa_circ_0082139 | exonic | chr7 | 0.000961569 | 0.005364199 | 1.5521693 | hsa_circ_0077527 | exonic | chr6 | 1.46066E-05 | 0.000567256 | 1.6198795 |
| hsa_circ_0027641 | exonic | chr12 | 0.000552625 | 0.003738618 | 1.5514809 | hsa_circ_0002069 | exonic | chr17 | 4.61555E-05 | 0.000929676 | 1.6098117 |
| hsa_circ_0001626 | exonic | chr6 | 0.001800834 | 0.008206728 | 1.5494381 | hsa_circ_0008305 | exonic | chr8 | 0.003283617 | 0.012504509 | 1.6078069 |
| hsa_circ_0000738 | exonic | chr17 | 9.80246E-05 | 0.001365225 | 1.5473397 | hsa_circ_0079375 | exonic | chr7 | 0.002039795 | 0.008916705 | 1.6062245 |
| hsa_circ_0007527 | exonic | chr15 | 0.00040475 | 0.003030504 | 1.5392491 | hsa_circ_0003838 | exonic | chr15 | 6.3161E-05 | 0.001065295 | 1.6042359 |
| hsa_circ_0092321 | intronic | chr9 | 0.000127542 | 0.001494495 | 1.537961 | hsa_circ_0000092 | intronic | chr1 | 0.000301087 | 0.002642505 | 1.6026659 |
| hsa_circ_0070356 | exonic | chr4 | 0.000272593 | 0.002519169 | 1.5295357 | hsa_circ_0000729 | intronic | chr16 | 0.000293954 | 0.002627453 | 1.6012241 |
| hsa_circ_0081343 | exonic | chr7 | 0.000794083 | 0.00470359 | 1.5286118 | hsa_circ_0071989 | exonic | chr5 | 0.000632729 | 0.003963053 | 1.5956346 |
| hsa_circ_0010358 | exonic | chr1 | 1.48682E-05 | 0.000567256 | 1.5230109 | hsa_circ_0000670 | intronic | chr16 | 0.000447097 | 0.00323947 | 1.5948105 |
| hsa_circ_0004896 | exonic | chr10 | 0.000344113 | 0.002847068 | 1.5226052 | hsa_circ_0000662 | intronic | chr16 | 3.53416E-06 | 0.000393416 | 1.5920095 |
| hsa_circ_0083377 | exonic | chr8 | 0.000788553 | 0.00470359 | 1.5217596 | hsa_circ_0047585 | exonic | chr18 | 1.67084E-05 | 0.000567256 | 1.588196 |
| hsa_circ_0006015 | exonic | chr18 | 1.76037E-05 | 0.000567256 | 1.5192455 | hsa_circ_0092360 | intronic | chr17 | 3.32191E-05 | 0.000767487 | 1.5863903 |
| hsa_circ_0058792 | exonic | chr2 | 0.000399368 | 0.003030504 | 1.516335 | hsa_circ_0007367 | exonic | chr9 | 0.000466278 | 0.003301322 | 1.5804673 |
| hsa_circ_0076054 | exonic | chr6 | 0.025872027 | 0.060343423 | 1.5118642 | hsa_circ_0001022 | intragenic | chr2 | 0.002775504 | 0.011034431 | 1.5798094 |
| hsa_circ_0039914 | exonic | chr16 | 9.97893E-05 | 0.001372817 | 1.5081694 | hsa_circ_0015454 | exonic | chr1 | 0.000351964 | 0.00287073 | 1.5796046 |
| hsa_circ_0000375 | exonic | chr12 | 4.70722E-05 | 0.000929676 | 1.5078233 | hsa_circ_0004928 | exonic | chr9 | 0.007872679 | 0.02416064 | 1.5662095 |
| hsa_circ_0014022 | exonic | chr1 | 3.0204E-05 | 0.000733668 | 1.5054967 | hsa_circ_0005281 | exonic | chr17 | 0.000248712 | 0.00236084 | 1.5654533 |
| hsa_circ_0018611 | exonic | chr10 | 0.005501281 | 0.018355092 | 1.5021766 | hsa_circ_0004552 | exonic | chr19 | 0.000300397 | 0.002642505 | 1.5643921 |
| hsa_circ_0008979 | exonic | chr2 | 1.45452E-05 | 0.000567256 | 1.5012409 | hsa_circ_0032254 | exonic | chr14 | 0.00132065 | 0.006612797 | 1.5567113 |
|  |  |  |  |  |  | hsa_circ_0006944 | exonic | chr7 | 0.006099585 | 0.019759105 | 1.5543956 |
|  |  |  |  |  |  | hsa_circ_0083756 | exonic | chr8 | 0.004394327 | 0.015529159 | 1.5344266 |
|  |  |  |  |  |  | hsa_circ_0002451 | exonic | chr7 | 8.65958E-05 | 0.001293129 | 1.5284137 |
|  |  |  |  |  |  | hsa_circ_0004136 | exonic | chr6 | 0.002377457 | 0.009786985 | 1.5279942 |
|  |  |  |  |  |  | hsa_circ_0092337 | intronic | chr16 | 0.000536072 | 0.003677423 | 1.514888 |
|  |  |  |  |  |  | hsa_circ_0007158 | exonic | chr5 | 3.97749E-05 | 0.000854461 | 1.5129586 |
|  |  |  |  |  |  | hsa_circ_0018004 | exonic | chr10 | 0.001369505 | 0.006775593 | 1.512753 |
|  |  |  |  |  |  | hsa_circ_0077930 | exonic | chr6 | 0.015965532 | 0.040879473 | 1.5113935 |
|  |  |  |  |  |  | hsa_circ_0003039 | exonic | chr1 | 1.10548E-05 | 0.000510816 | 1.507587 |
|  |  |  |  |  |  | hsa_circ_0047151 | exonic | chr18 | 0.000955939 | 0.005357196 | 1.504004 |
|  |  |  |  |  |  | hsa_circ_0088072 | exonic | chr9 | 0.001067257 | 0.005735796 | 1.5021611 |
