# Supplementary material for: Circular RNA hsa_circ_0008305 (circPTK2) inhibits TGF-β-induced epithelial-mesenchymal transition and metastasis by controlling TIF1γ in non-small cell lung cancer
Source: Mol Cancer. 2018 Sep 27;17:140. doi: 10.1186/s12943-018-0889-7 (PMC6161470; doi:10.1186/s12943-018-0889-7)
Supplement: Supplementary file 4 — Table S2. Sequences for construction of luciferase reporter plasmids containing predicted miR-429 and miR-200b-3p target sites in TIF1γ 3’-UTR and circPTK2. (DOC 38 kb) [file 12943_2018_889_MOESM4_ESM.doc]

**Table S2. Sequences for construction of luciferase reporter plasmids containing predicted miR-429 and miR-200b-3p target sites in *TIF1γ* 3'-UTR and circPTK2**

| Name | Target site | Sequences (5'—3') ^*^ |
| --- | --- | --- |
| *TIF1γ* 3'-UTR-1 | 145-152 | TGGGCACCTCCTTGAAGAAGCTGATAGCTTTTACA**CAGTATT**AGATTGAAATAATGGACAGAA |
| Mutant |  | TGGGCACCTCCTTGAAGAAGCTGATAGCTTTTACA**GTCATAA**AGATTGAAATAATGGACAGAA |
|  |  |  |
| *TIF1γ* 3'-UTR-2 | 2247-2253 | AACTGAGGGGAGAAACATTTTAAGTAAATATTTTT**CAGTATT**TGGGGCCTTAAAAAATAATTG |
| Mutant |  | AACTGAGGGGAGAAACATTTTAAGTAAATATTTTT**GTCATAA**TGGGGCCTTAAAAAATAATTG |
|  |  |  |
| *TIF1γ* 3'-UTR-3 | 2690-2696 | ATACTCTCTCACATTTGTATTTGTTTTTTAAATGG**CAGTATT**TTAGAAGATTTGGAGAAAAGT |
| Mutant |  | ATACTCTCTCACATTTGTATTTGTTTTTTAAATGG**GTCATAA**TTAGAAGATTTGGAGAAAAGT |
|  |  |  |
| *TIF1γ* 3'-UTR-4 | 4486-4492 | GGGTTAAATTCACAGCCTTACTAGTTCCTTGCTTC**CAGTATT**TCAATTGGTCTCCTCCCCTCA |
| Mutant |  | GGGTTAAATTCACAGCCTTACTAGTTCCTTGCTTC**GTCATAA**TCAATTGGTCTCCTCCCCTCA |
|  |  |  |
| circPTK2 | 314-320 | ACATCTTGCTGACTTCACTCAAGTGCAAACCATT**CAGTATT**CAAACAGTGAAGACAAGGAC |
| Mutant |  | ACATCTTGCTGACTTCACTCAAGTGCAAACCATT**GTCATAA**CAAACAGTGAAGACAAGGAC |

^*^ Underlined: predicted target sites of miR-429 and miR-200b-3p by TargetScan/miRBase and miRanda.
