# Supplementary material for: Circular RNA hsa_circ_0008305 (circPTK2) inhibits TGF-β-induced epithelial-mesenchymal transition and metastasis by controlling TIF1γ in non-small cell lung cancer
Source: Mol Cancer. 2018 Sep 27;17:140. doi: 10.1186/s12943-018-0889-7 (PMC6161470; doi:10.1186/s12943-018-0889-7)
Supplement: Supplementary file 10 — Table S3. Demographic and clinical characteristics of 73 NSCLC patients and relative expression of circPTK2 and TIF1γ mRNA in 73 paired NSCLC tissues. (DOC 134 kb) [file 12943_2018_889_MOESM10_ESM.doc]

**Table S3. Demographic and clinical characteristics of 73 NSCLC patients and relative expression of circPTK2 and TIF1γ mRNA in 73 paired NSCLC tissues**

| Case | Age (years) | Histology ^*^ | TNM | Stage | circPTK2 (T/N) ^†^ | TIF1γ mRNA (T/N) ^†^ |
| --- | --- | --- | --- | --- | --- | --- |
| 1 | 70 | SqC | T3N1M0 | IIIA | 0.2134 | 1.4635 |
| 2 | 65 | AdC | T2N2M0 | IIIA | 0.6487 | 0.7219 |
| 3 | 73 | AdC | T1N0M0 | IA | 0.4277 | 0.6573 |
| 4 | 57 | AdC | T2N1M0 | IIA | 0.2359 | 0.8796 |
| 5 | 65 | AdC | T2N1M0 | IIA | 0.6741 | 0.6953 |
| 6 | 64 | SqC | T1N0M0 | IA | 1.4618 | 2.2939 |
| 7 | 60 | SqC | T2N1M0 | IIA | 0.0942 | 1.0471 |
| 8 | 63 | Other | T4N0M1 | IV | 0.0831 | 0.1708 |
| 9 | 67 | AdC | T4N0M0 | IIIA | 0.4398 | 0.8466 |
| 10 | 65 | AdC | T1N0M0 | IA | 1.3457 | 1.0692 |
| 11 | 35 | AdC | T2N2M0 | IIIA | 0.0385 | 0.3400 |
| 12 | 77 | AdC | T1N2M0 | IIIA | 1.0202 | 0.9727 |
| 13 | 69 | AdC | T2N2M0 | IIIA | 0.0952 | 0.7759 |
| 14 | 72 | AdC | T2N0M0 | IIA | 0.0654 | 1.2604 |
| 15 | 58 | AdC | T3N0M0 | IIB | 1.3993 | 1.4845 |
| 16 | 72 | SqC | T2N2M0 | IIIA | 0.3044 | 0.2085 |
| 17 | 87 | AdC | T2N0M0 | IB | 0.2641 | 0.3543 |
| 18 | 85 | Other | T2N0M0 | IB | 0.0887 | 0.3880 |
| 19 | 64 | SqC | T2N2M0 | IIIA | 0.3511 | 0.4683 |
| 20 | 55 | SqC | T1N2M0 | IIIA | 0.3276 | 0.8442 |
| 21 | 62 | AdC | T2N1M0 | IIA | 0.5251 | 0.3770 |
| 22 | 57 | AdC | T2N1M0 | IIA | 0.2600 | 0.6273 |
| 23 | 63 | Other | T4N0M1 | IV | 0.0438 | 0.1948 |
| 24 | 54 | AdC | T1N0M0 | IA | 0.6532 | 0.6327 |
| 25 | 72 | AdC | T2N0M0 | IB | 1.8826 | 1.7546 |
| 26 | 71 | AdC | T2N0M0 | IIA | 0.1054 | 0.6571 |
| 27 | 60 | AdC | T2N2M0 | IIIA | 0.2103 | 0.7195 |
| 28 | 59 | AdC | T4N2M0 | IIIB | 0.1675 | 0.8958 |
| 29 | 40 | AdC | T1N1M0 | IIA | 0.1316 | 0.3789 |
| 30 | 78 | AdC | T4N1M0 | IIIA | 0.6455 | 1.8925 |
| 31 | 74 | SqC | T2N2M0 | IIIA | 0.2328 | 0.2508 |
| 32 | 63 | AdC | T1N1M0 | IIA | 0.2884 | 0.7395 |
| 33 | 71 | AdC | T2N0M0 | IB | 0.1040 | 0.4603 |
| 34 | 58 | AdC | T4N0M1 | IV | 0.0813 | 1.4031 |
| 35 | 73 | Other | T1N0M0 | IA | 0.2206 | 0.6715 |
| 36 | 67 | SqC | T2N0M0 | IIA | 1.5580 | 1.1932 |
| 37 | 48 | AdC | T2N0M0 | IB | 0.1792 | 0.8810 |
| 38 | 65 | AdC | T1N0M0 | IA | 0.2449 | 0.5004 |
| 39 | 66 | AdC | T1N0M1 | IV | 0.6308 | 1.8532 |
| 40 | 58 | SqC | T2N1M0 | IIA | 0.2176 | 0.7338 |
| 41 | 68 | AdC | T4N0M0 | IIIA | 0.1042 | 0.2263 |
| 42 | 67 | AdC | T2N0M0 | IIA | 0.0708 | 0.8150 |
| 43 | 62 | AdC | T1N2M0 | IIIA | 0.3300 | 1.2839 |
| 44 | 60 | AdC | T2N2M0 | IIIA | 0.2577 | 1.6472 |
| 45 | 70 | SqC | T1N0M0 | IA | 0.3648 | 1.4689 |
| 46 | 57 | AdC | T2N0M0 | IB | 0.2400 | 0.9267 |
| 47 | 64 | AdC | T4N2M1 | IV | 0.1138 | 0.2852 |
| 48 | 68 | AdC | T1N1M0 | IIA | 0.0451 | 0.4049 |
| 49 | 65 | AdC | T2N0M0 | IIA | 0.2813 | 0.8590 |
| 50 | 36 | Other | T1N1M0 | IIA | 0.3182 | 0.3195 |
| 51 | 75 | AdC | T2N0M0 | IB | 1.0855 | 2.4856 |
| 52 | 69 | AdC | T2N0M0 | IB | 0.5895 | 1.7402 |
| 53 | 61 | SqC | T1N0M0 | IA | 0.6396 | 0.7658 |
| 54 | 61 | SqC | T2N1M0 | IIA | 0.2916 | 0.8408 |
| 55 | 77 | Other | T3N0M0 | IIB | 0.1892 | 0.4247 |
| 56 | 68 | SqC | T4N2M0 | IIIB | 0.1364 | 0.3055 |
| 57 | 68 | AdC | T2N0M0 | IB | 0.8999 | 2.0114 |
| 58 | 58 | Other | T3N0M0 | IIB | 0.2178 | 0.7934 |
| 59 | 73 | SqC | T3N0M0 | IIB | 0.1712 | 0.2906 |
| 60 | 71 | AdC | T2N0M0 | IB | 0.5972 | 1.0742 |
| 61 | 47 | Other | T3N2M0 | IIIA | 0.8962 | 1.3243 |
| 62 | 65 | AdC | T1N2M0 | IIIA | 1.4620 | 0.6287 |
| 63 | 60 | AdC | T1N2M0 | IIIA | 0.6381 | 1.8595 |
| 64 | 57 | AdC | T2N2M0 | IIIA | 0.7873 | 0.2051 |
| 65 | 64 | Other | T4N2M0 | IIIB | 0.0975 | 0.2526 |
| 66 | 39 | AdC | T1N0M0 | IA | 0.2682 | 0.2933 |
| 67 | 54 | SqC | T4N1M0 | IIIA | 0.2597 | 0.3737 |
| 68 | 70 | AdC | T4N0M1 | IV | 0.6010 | 0.9233 |
| 69 | 53 | AdC | T2N0M0 | IB | 0.4009 | 0.8821 |
| 70 | 64 | AdC | T1N0M0 | IA | 1.8881 | 0.9259 |
| 71 | 60 | AdC | T4N0M1 | IV | 0.4796 | 0.6974 |
| 72 | 65 | AdC | T2N1M0 | IIA | 0.7888 | 0.8497 |
| 73 | 47 | Other | T4N2M1 | IV | 0.1475 | 1.1763 |

^*^ AdC, adenocarcinoma; SqC, squamous cell carcinoma; Other, large cell carcinoma, etc.

^†^ T, NSCLC tissues; N, paired noncancerous lung tissues. Ratio values of T/N > 1.0, T/N < 1.0 and T/N ≈ 1.0 represent increased, reduced and preserved expression, respectively.
