# Supplementary material for: Circular RNA hsa_circ_0008305 (circPTK2) inhibits TGF-β-induced epithelial-mesenchymal transition and metastasis by controlling TIF1γ in non-small cell lung cancer
Source: Mol Cancer. 2018 Sep 27;17:140. doi: 10.1186/s12943-018-0889-7 (PMC6161470; doi:10.1186/s12943-018-0889-7)
Supplement: Supplementary file 12 — Table S4. Demographic and clinical characteristics of 73 NSCLC patients and relative expression of miR-429 and miR-200b-3p in 73 paired NSCLC tissues. (DOC 151 kb) [file 12943_2018_889_MOESM12_ESM.doc]

**Table S4. Demographic and clinical characteristics of 73 NSCLC patients and relative expression of miR-429 and miR-200b-3p in 73 paired NSCLC tissues**

| Case | Age (years) | Histology ^*^ | TNM | Stage | miR-429 (T/N) ^†^ | miR-200b-3p (T/N) ^†^ |
| --- | --- | --- | --- | --- | --- | --- |
| 1 | 70 | SqC | T3N1M0 | IIIA | 43.7118 | 1.3863 |
| 2 | 65 | AdC | T2N2M0 | IIIA | 8.7232 | 5.4077 |
| 3 | 73 | AdC | T1N0M0 | IA | 2.5394 | 2.0207 |
| 4 | 57 | AdC | T2N1M0 | IIA | 0.3026 | 0.6434 |
| 5 | 65 | AdC | T2N1M0 | IIA | 0.5271 | 1.6999 |
| 6 | 64 | SqC | T1N0M0 | IA | 0.4400 | 1.3488 |
| 7 | 60 | SqC | T2N1M0 | IIA | 5.8164 | 4.0423 |
| 8 | 63 | Other | T4N0M1 | IV | 0.3275 | 0.4444 |
| 9 | 67 | AdC | T4N0M0 | IIIA | 1.4340 | 1.5382 |
| 10 | 65 | AdC | T1N0M0 | IA | 0.7071 | 0.7426 |
| 11 | 35 | AdC | T2N2M0 | IIIA | 2.0395 | 3.7445 |
| 12 | 77 | AdC | T1N2M0 | IIIA | 0.9693 | 0.9659 |
| 13 | 69 | AdC | T2N2M0 | IIIA | 2.0347 | 2.7321 |
| 14 | 72 | AdC | T2N0M0 | IIA | 1.3524 | 1.4785 |
| 15 | 58 | AdC | T3N0M0 | IIB | 8.6114 | 4.5315 |
| 16 | 72 | SqC | T2N2M0 | IIIA | 22.7109 | 3.4158 |
| 17 | 87 | AdC | T2N0M0 | IB | 7.6610 | 4.7174 |
| 18 | 85 | Other | T2N0M0 | IB | 0.2921 | 0.7201 |
| 19 | 64 | SqC | T2N2M0 | IIIA | 0.4305 | 0.5087 |
| 20 | 55 | SqC | T1N2M0 | IIIA | 3.0087 | 2.2013 |
| 21 | 62 | AdC | T2N1M0 | IIA | 26.1026 | 36.6362 |
| 22 | 57 | AdC | T2N1M0 | IIA | 1.1096 | 3.1252 |
| 23 | 63 | Other | T4N0M1 | IV | 1.1735 | 2.9237 |
| 24 | 54 | AdC | T1N0M0 | IA | 5.7168 | 10.1530 |
| 25 | 72 | AdC | T2N0M0 | IB | 5.5047 | 0.4475 |
| 26 | 71 | AdC | T2N0M0 | IIA | 16.4310 | 18.2539 |
| 27 | 60 | AdC | T2N2M0 | IIIA | 12.7814 | 3.1696 |
| 28 | 59 | AdC | T4N2M0 | IIIB | 0.2078 | 0.1017 |
| 29 | 40 | AdC | T1N1M0 | IIA | 0.0957 | 0.0308 |
| 30 | 78 | AdC | T4N1M0 | IIIA | 0.6950 | 0.8020 |
| 31 | 74 | SqC | T2N2M0 | IIIA | 0.3649 | 0.3778 |
| 32 | 63 | AdC | T1N1M0 | IIA | 2.2890 | 4.9932 |
| 33 | 71 | AdC | T2N0M0 | IB | 4.6767 | 1.0391 |
| 34 | 58 | AdC | T4N0M1 | IV | 53.6363 | 45.9848 |
| 35 | 73 | Other | T1N0M0 | IA | 1.8816 | 0.8086 |
| 36 | 67 | SqC | T2N0M0 | IIA | 0.4141 | 0.1402 |
| 37 | 48 | AdC | T2N0M0 | IB | 42.1126 | 22.5493 |
| 38 | 65 | AdC | T1N0M0 | IA | 0.4830 | 0.2406 |
| 39 | 66 | AdC | T1N0M1 | IV | 4.2701 | 12.6787 |
| 40 | 58 | SqC | T2N1M0 | IIA | 1.2224 | 2.3127 |
| 41 | 68 | AdC | T4N0M0 | IIIA | 4.0796 | 1.4271 |
| 42 | 67 | AdC | T2N0M0 | IIA | 1.0861 | 0.8439 |
| 43 | 62 | AdC | T1N2M0 | IIIA | 0.3522 | 1.9325 |
| 44 | 60 | AdC | T2N2M0 | IIIA | 0.2253 | 0.1975 |
| 45 | 70 | SqC | T1N0M0 | IA | 0.1432 | 0.2111 |
| 46 | 57 | AdC | T2N0M0 | IB | 1.6036 | 0.2570 |
| 47 | 64 | AdC | T4N2M1 | IV | 1.1129 | 0.4479 |
| 48 | 68 | AdC | T1N1M0 | IIA | 1.8419 | 0.6782 |
| 49 | 65 | AdC | T2N0M0 | IIA | 3.8957 | 3.7179 |
| 50 | 36 | Other | T1N1M0 | IIA | 4.9015 | 3.6188 |
| 51 | 75 | AdC | T2N0M0 | IB | 0.9284 | 1.0900 |
| 52 | 69 | AdC | T2N0M0 | IB | 5.7154 | 0.8783 |
| 53 | 61 | SqC | T1N0M0 | IA | 0.4076 | 0.3186 |
| 54 | 61 | SqC | T2N1M0 | IIA | 1.9275 | 1.0538 |
| 55 | 77 | Other | T3N0M0 | IIB | 0.0591 | 0.0712 |
| 56 | 68 | SqC | T4N2M0 | IIIB | 9.2468 | 0.3294 |
| 57 | 68 | AdC | T2N0M0 | IB | 15.0869 | 13.6421 |
| 58 | 58 | Other | T3N0M0 | IIB | 3.0959 | 1.8600 |
| 59 | 73 | SqC | T3N0M0 | IIB | 0.0574 | 0.0522 |
| 60 | 71 | AdC | T2N0M0 | IB | 7.5226 | 5.8457 |
| 61 | 47 | Other | T3N2M0 | IIIA | 0.2214 | 0.5762 |
| 62 | 65 | AdC | T1N2M0 | IIIA | 4.3913 | 4.2428 |
| 63 | 60 | AdC | T1N2M0 | IIIA | 2.0859 | 2.5564 |
| 64 | 57 | AdC | T2N2M0 | IIIA | 0.3025 | 0.3649 |
| 65 | 64 | Other | T4N2M0 | IIIB | 0.5881 | 0.5007 |
| 66 | 39 | AdC | T1N0M0 | IA | 0.3512 | 0.3476 |
| 67 | 54 | SqC | T4N1M0 | IIIA | 0.1014 | 0.1901 |
| 68 | 70 | AdC | T4N0M1 | IV | 60.6960 | 42.7893 |
| 69 | 53 | AdC | T2N0M0 | IB | 32.5762 | 29.7220 |
| 70 | 64 | AdC | T1N0M0 | IA | 48.6078 | 148.0561 |
| 71 | 60 | AdC | T4N0M1 | IV | 0.3846 | 0.4831 |
| 72 | 65 | AdC | T2N1M0 | IIA | 0.5226 | 0.3118 |
| 73 | 47 | Other | T4N2M1 | IV | 11.5978 | 15.3968 |

^*^ AdC, adenocarcinoma; SqC, squamous cell carcinoma; Other, large cell carcinoma, etc.

^†^ T, NSCLC tissues; N, paired noncancerous lung tissues. Ratio values of T/N > 1.0, T/N < 1.0 and T/N ≈ 1.0 represent increased, reduced and preserved expression, respectively.
