# Supplementary material for: Circular RNA hsa_circ_0008305 (circPTK2) inhibits TGF-β-induced epithelial-mesenchymal transition and metastasis by controlling TIF1γ in non-small cell lung cancer
Source: Mol Cancer. 2018 Sep 27;17:140. doi: 10.1186/s12943-018-0889-7 (PMC6161470; doi:10.1186/s12943-018-0889-7)
Supplement: Supplementary file 16 — Table S5. Primers for qRT-PCR analysis. (DOC 31 kb) [file 12943_2018_889_MOESM16_ESM.doc]

**Table S5. Primers for qRT-PCR analysis**

| Name | Sequences (5'—3') |
| --- | --- |
| RT primers  miR-429  miR-200b-3p  U6  qRT-PCR primers^*^  miR-429  miR-200b-3p  U6  TIF1γ  SNAIL  PTK2  β-actin  CircPTK2        GAPDH | GTTGGCTCTGGTGCAGGGTCCGAGGTATTCGCACCAGAGCCAACACGGTTTT  GTTGGCTCTGGTGCAGGGTCCGAGGTATTCGCACCAGAGCCAACTCATCATT  CGAGCACAGAATCGCTTCACGAATTTGCGTGTCAT  F: GTGCAGGGTCCGAGGTATT; R: GCCGTCGTAATACTGTCTGGT  F: GTGCAGGGTCCGAGGTATT; R: GCCGTCGTAATACTGCCTGGT  F: CGAGCACAGAATCGCTTCA; R: CTCGCTTCGGCAGCACATAT  F: AGCAACGGCGACATCCA; R: TGCATTCTTGGCGGCATA  F: CGAAAGGCCTTCAACTGCAAAT; R: ACTGGTACTTCTTGACATCTG  F: ACATTATTGGCCACTGTGGATGAG; R: GGGCCAGTTTCATCTTGTTGATGAG  F: CACAGAGCCTCGCCTTTGCC; R: ACCCATGCCCACCATCACG  Divergent primers:  F: GCCAACAGCGAAAAGCAAG; R: TTTGGCCTTGACAGAATCCAG  Convergent primers:  F: GCCAAAACACTAAGAAAACTGATCC; R: ACCAAGAGCACACTTGAAGCAT  Divergent primers:  F: TCCCCCACCACACTGAATCT; R: AACAGGAGGAGCAGAGAGCG  Convergent primers:  F: CCTGCCGTCTAGAAAAACCTG; R: AGTGGGTGTCGCTGTTGAAGT |

^*^ F, forward; R, reverse.
